# Five New Alkaloids from *Cephalotaxus lanceolata* and *C. fortunei var. alpina*

**DOI:** 10.1007/s13659-016-0093-7

**Published:** 2016-03-26

**Authors:** Ling Ni, Xiu-Hong Zhong, Jie Cai, Mei-Fen Bao, Bing-Jie Zhang, Jing Wu, Xiang-Hai Cai

**Affiliations:** 1 State Key Laboratory of Phytochemistry and Plant Resources in West China, Kunming Institute of Botany, Chinese Academy of Sciences, Kunming, 650201 People’s Republic of China; 2 University of Chinese Academy of Sciences, Beijing, 100039 People’s Republic of China; 3 Germplasm Bank of Wild Species in Southwest China, Kunming Institute of Botany, Chinese Academy of Sciences, Kunming, 650201 People’s Republic of China

**Keywords:** *Cephalotaxus*, Alkaloids, Cytotoxicity

## Abstract

Five new alkaloids (**1**–**5)** were isolated from the leaves and twigs of *Cephalotaxus lanceolata* and *C. fortunei var. alpina* along with 24 known alkaloids. The new structures were elucidated based on spectroscopic data including 1D and 2D NMR, FTIR, UV and MS. These new alkaloids showed no cytotoxicity to HeLa, SGC-7901 gastric cancer, and A-549 lung cancer cell lines.

**Electronic supplementary material:**

The online version of this article (doi:10.1007/s13659-016-0093-7) contains supplementary material, which is available to authorized users.

## Introduction

**Fig. 1:**
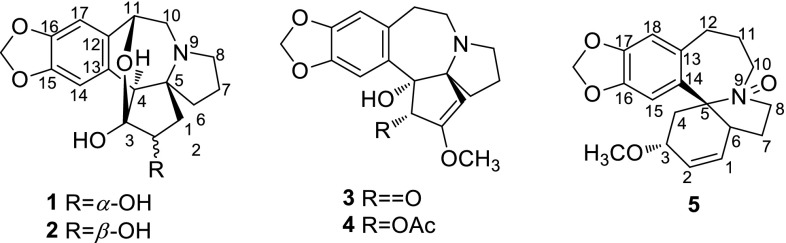
Structures of alkaloids from *C. lanceolata* and *C. fortunei var. alpine*

Various constituents of *Cephalotaxus* genus have been reported, including alkaloids [1–6], tropones [7–10], lignans [10, 11], diterpenes [9], flavonoids [6, 10]. Previous investigations led to approximate 100 *Cephalotaxus* alkaloids, which were mainly classified into two structural types, i.e., homoerythrina and cephalotaxine-type, and the latter demonstrated remarkable antitumor activities [12]. For example, homoharringtonine among cephalotaxine alkaloids was successfully used to treat acute leukemia. As for homoharringtonine, the side chains played an important role in the anticancer activity of these compounds which possessed H-3 *α*-configuration. So far only reported cephalezomines G possessed H-3 *β*-configuration. Both homoerythrina and cephalotaxine had same biogenetic origin. However, most of homoerythrinas almost with H-3 *α*-configuration reminded us that there were more cephalotaxines with same configuration. As a part of our continuous research for *Cephalotaxus* alkaloids, five new alkaloids, together with 24 known ones (Fig. 1) were isolated from leaves and twigs of *C. lanceolata* and *C. fortunei var. alpina*. The known alkaloids were identified as drupacine (**6**) [2], cephalotaxinone (**7**) [13], acetycephalotaxine (**8**) [14], cephalezomine J (**9**) [5], desmethylcephalotaxine (**10**) [15], isocephalotaxinone (**11**) [16], 1l-hydroxycephalotaxin (**12**) [2], cephalotaxine (**13**) [17], lucidinine (**14**) [18], comosidine (**15**) [18], schelhammeridine (**16**) [19], 3-epischelhammeridine (**17**) [20], comosine (**18**) [21], 3-epicomosine (**19**) [20], 3-epischelhammericine (**20**) [20], fortunine (**21**) [22], taxodine (**22**) [23], *O*-methylschlammericine (**23**) [13], cephalezomine M (**24**) [5], homoisoharringtonine (**25**) [24], homoharingtonine (**26**) [25], isoharringtonine (**27**) [25, 26], epidesoxyharringtonine (**28**) [27], desoxyharringtonine (**29**) [28] by comparison with literatures.

## Results and Discussion

**Fig. 2:**
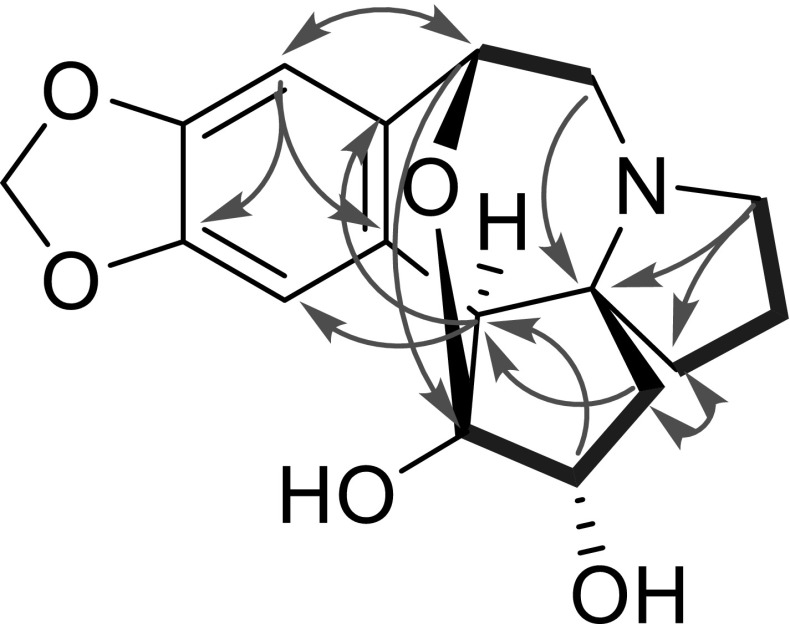
Key ^1^H–^1^H COSY () and HMBC () correlations of compound **1**. (Color figure online)

Newly isolates (**1**–**5**) probably belong to alkaloids as they exhibited a positive reaction with Dragendorff’s reagent. Alkaloid **1** was isolated as white powder. Its UV absorption bands at 203 and 291 nm and IR absorption bands at 3520, 3406, 1631, 1500, 1482, 1342 cm^−1^ were consistent with those of *Cephalotaxus* alkaloids [2]. Analysis of the ^1^H and ^13^C NMR data of **1** (Tables 1, 2) revealed several typical functionalities similar to those of the known alkaloid drupacine (**6**) [2], including a tetrasubstituted benzene ring with two *para* H-atoms (*δ*_H_ 6.76, *δ*_C_ 110.1; *δ*_H_ 6.72, *δ*_C_ 106.0; *δ*_C_ 128.6, 132.4, 146.8, 147.4), a –OCH_2_O– moiety (*δ*_H_ 5.97; *δ*_C_ 101.5), a ketal carbon (*δ*_C_ 106.7), two *O*-bearing CH groups (*δ*_H_ 3.86, *δ*_C_ 76.7; *δ*_H_ 4.81, *δ*_C_ 76.1), and two –OH groups (*δ*_H_ 3.53 and 4.68). The molecular formula of **1** was established as C_17_H_19_NO_5_ with nine degrees of unsaturation by HRESIMS ([M+H]^+^ at *m/z* 318.1336), absence of a methyl than that of **6**. The HMBC correlations (Fig. 2) of the methine signal (*δ*_H_ 4.81) with C-12 (*δ*_C_ 132.4), C-13 (*δ*_C_ 128.6), and C-17 (*δ*_C_ 106.0) allowed its position as C-11. Likewise, the other signal *δ*_H_ 3.38 was assigned to CH-4 based on its HMBC correlations with *δ*_C_ 110.1 (C-14), C-12 and *δ*_C_ 39.2 (C-6). The obvious HMBC correlation between methylene protons (*δ*_H_ 1.37 and 2.23) with C-6 and C-3 attributed it to C-1. The proton signal *δ*_H_ 3.86 was assigned to H-2 based on its correlation with *δ*_H_ 2.23 in the ^1^H–^1^H COSY (Fig. 2) spectrum. The ketal carbon (*δ*_C_ 106.7) was located at C-3 by its HMBC correlations from H-1, 2 and 4. The HMBC crosspeak of H-11/C-3 showed an oxygen bridge between C-11/C-1 in **1** consistent with its degrees of unsaturation. H-2 was established as *β*-orientation on the basis of the coupling constant (d, *J* = 6.4 Hz) of H-2. Consequently, the structure of **1** was confirmed as shown in Fig. 1, and named cephalotine A.

**Table 1:** ^1^H NMR spectroscopic data of **1**–**5** (*δ* in ppm and *J* in Hz)

Position	*δ*_H_(**1**)^a^	*δ*_H_(**2**)^a^	*δ*_H_(**3**)^b^	*δ*_H_(**4**)^a^	*δ*_H_(**5**)^a^
1	1.37 d (15.0) 2.23 dd (15.0, 6.4)	1.70 dd (14.4, 8.4) 1.82 dd (14.4, 9.6)	6.69 s	5.21 s	6.12 m
2	3.86 d (6.4)	4.05 t (8.9)			5.74 d (10.2)
3			–	5.47 s	2.88 overlap
4	3.38 s	3.10 s	–	–	1.66 overlap 2.92 overlap
6	1.67 overlap 1.69 overlap	1.58 overlap 1.68 m	1.79 m 2.20 dd (11.4, 4.2)	1.65 overlap 2.30 m	3.93 overlap
7	1.55 overlap 1.57 overlap	1.56 overlap 1.58 overlap	1.64 m 1.82 m	1.49 overlap 1.63 overlap	1.70 overlap 2.64 m
8	2.28 m 2.60 td (8.8, 3.6)	2.25 m 2.59 m	2.80 m 2.95 overlap	2.67 overlap 2.95 m	3.54 td (2.4, 13.2) 4.08 m
10	2.55 d (12.3) 2.71 dd (12.3, 4.0)	2.65 d (12.2) 2.71 dd (12.2, 4.0)	2.61 dd (11.4, 7.8) 2.75 m	2.65 overlap 2.70 m	3.43 d (13.0) 3.92 overlap
11	4.81 d (3.1)	4.87 d (3.8)	2.37 m 2.49 dd (15.0, 7.8)	2.36 dd (14.4, 6.8) 3.12 m	1.68 overlap 2.57 m
12					2.77 dd (5.8, 15.7) 3.31 td (2.3, 11.2)
14	6.76 s	6.73 s	7.31 s	7.15 s	
15					6.62 s
17	6.72 s	6.74 s	6.63 s	6.67 s	
18					6.67 s
OCH_2_O	5.97 s 5.93 s	5.93 d (1.1) 5.97 d (1.1)	5.96 s 5.97 s	5.93 s 5.96 s	5.93 s 5.94 s
2-OH	3.53 br*	3.45 br*	–		
3-OH	4.68 br*	4.65 br*	–		
4-OH	–	–	5.11		
2-OCH_3_	–	–	3.81 s	3.66 s	
3-OCH_3_					3.22 s
CH_3_CO	–	–	–	2.51 s	

Alkaloids **1**, **2**, **4** and **5** recorded in acetone-*d*_6_; **3** in DMSO-*d*_6_

* Assignments may be interchanged

^a^Recorded at 400 MHz

^b^Recorded at 600 MHz

**Table 2:** ^13^C NMR spectroscopic data of alkaloids **1**–**5** (*δ* in ppm)

Position	*δ*_C_(**1**)^a^	*δ*_C_(**2**)^a^	*δ*_C_(**3**)^b^	*δ*_C_(**4**)^a^	*δ*_C_(**5**)^a^
1	34.2 t	34.2 t	125.0 d	101.2 d	130.0 d
2	76.7 d	77.0 d	158.1 s	155.2 s	128.7 d
3	106.7 s	104.0 s	200.1 s	83.1 s	76.0 d
4	53.7 d	55.1 d	81.9 s	86.1 s	31.6 t
5	67.4 s	65.5 s	71.0 s	76.8 s	87.0 s
6	39.2 t	38.6 t	34.0 t	35.8 t	43.3 d
7	20.6 t	20.1 t	20.0 t	20.1 t	27.1 t
8	50.2 t	50.1 t	54.1 t	54.2 t	67.6 t
10	55.1 t	54.9 t	47.8 t	48.1 t	63.3 t
11	76.1 d	76.2 d	32.4 t	31.0 t	23.0 t
12	132.4 s	132.4 s	130.7 s	131.8 s	35.6 t
13	128.6 s	128.5 s	134.3 s	133.4 s	135.0 s
14	110.1 d	110.0 d	108.2 d	108.0 d	128.2 s
15	147.4 s	146.8 s	146.7 s	144.9 s	111.1d
16	146.8 s	147.3 s	147.4 s	145.6 s	146.9 s
17	106.7 d	106.0 d	110.1 d	109.2 d	148.0 s
18					112.7 d
OCH_2_O	101.5 t	101.5 t	101.8 t	100.6 t	102.4 t
2-OCH_3_	–	–	57.5 q	56.9 q	
3-OCH_3_					56.1 q
CH_3_ CO	–	–	–	168.9 s	
CH_3_CO				20.2 q	

Alkaloids **1**, **2**, **4** and **5** recorded in acetone-*d*_*6*_; **3** in DMSO-*d*_*6*_

^a^Recorded at 100 MHz

^b^Recorded at 150 MHz

Alkaloid **2** had the same molecular formula (HRESIMS *m/z* 318.1335 [M+H]^+^) and very similar UV and IR spectra as **1**. Comparison of the ^13^C NMR data of **2** and **1** (Table 2) suggested that both compounds shared the same planar structure. In the ^1^H NMR spectrum (Table 1), obvious difference between both alkaloids was that a proton signal *δ*_H_ 3.86 (d, *J* = 6.4 Hz, H-2) in **1** was replaced by *δ*_H_ 4.05 (t, *J* = 8.9 Hz) in **2**. This indicated *α*-configuration of H-2 in **2**, and confirmed by a ROESY correlation from H-2 to H-4. Thus, **2** was established as 3-epicephalotine A and named cephalotine B.

Alkaloid **3** displayed similar ^1^H and ^13^C NMR data (Tables 1, 2) to the known alkaloid cephalotaxinone (**7**) [13] except that a quaternary carbon (*δ*_C_ 81.9) in **3** substituted a methine in **7**. In addition, the HMBC correlations of both H-1 and H-14 with *δ*_C_ 81.9 located the quaternary carbon to C-4. The molecular formula C_18_H_19_NO_5_ of **3** from HRESIMS *m/z* at 330.1337 [M+H]^+^, 16 mass units higher than that of **7**, further indicated that **3** was an 4-hydroxy cephalotaxinone. Alkaloid **4** showed the similar ^13^C NMR data to the known alkaloid acetycephalotaxine (**8**) [14], except that a methine signal of **8** was substituted by a quaternary carbon *δ*_C_ 86.1 (s) in **4**. Like in **3**, the additional hydroxyl of **4** was also located at C-4 by its molecular formula C_20_H_23_NO_6_ by HRESIMS at *m/z* 374.1604 [M+H]^+^), 16 mass units higher than that of **8**. Further, this was supported by the HMBCs of *δ*_H_ 5.21 (H-1) and *δ*_H_ 7.15 (H-14) with *δ*_C_ 86.1 (C-4). The hydroxyl of **3** and **4** adopted *α*-orientation by the molecular model. The configuration of H-3 in both alkaloids was *α*-oriented by ROESY correlation between H-3 and H-11. Therefore, **3** and **4** were named cephalotines C and D, respectively.

Six methylenes, 3 methines, a methyoxyl and 5 quaternary carbons in the ^13^C NMR spectrum of alkaloid **5** revealed that **5** belongs to homoerythrina-type alkaloids rather than cephalotaxine-type alkaloids [2]. The ^13^C NMR and DEPT data of alkaloid **5** were similar to those of comosine (**18**) [21] with exception for three downfielded signals [87.0 (s), 67.6 (t), 63.3 (t)], suggesting a *N*-oxide moiety. Additionally, its molecular formula C_20_H_23_NO_4_ by HRESIMS (*m/z* 330.1717 [M+H]^+^) could support this presumption. The H-3 was allowed at *β*-configuration through ROESY correlations of H-3 with H-10 and H-12. Thus **5** was named as cephalotine E.

None of these compounds showed any significant activity against HeLa, SGC-7901 gastric cancer, and A-549 lung cancer cell lines (IC_50_ > 20 *μ*M).

## Experimental Section

### General Experimental Procedures

Optical rotations were carried out using a Horiba SEPA-300 polarimeter and *J*ASCO DIP-370 digital polarimeter. UV spectra were recorded on Shimadzu 2401Aspectrophotometer. IR Spectra were obtained on Brucker Tensor 27 infrared spectrophotometer with KBr pellets. ^1^H, ^13^C and 2D NMR spectral data were measured on a Bruker Avance III-600, DRX-500, and AM-400 MHz spectrometers with SiMe_4_ as an internal standard. HRESIMS data were recorded on an Agilent G6230 TOF MS. Column chromatography (CC) was performed with silica gel (200–300 mesh, Qing-dao Haiyang Chemical Co., Ltd., Qingdao, China). RP-18 silica gel (20–45 *μ*m, Fuji Silysia Chemical Ltd., Japan). Fractions were monitored by TLC on silica gel plates (GF254, Qingdao Haiyang Chemical Co., Ltd.) and spots visualized with Dragendorff’s reagent spray. MPLC was employed using a Buchi pump system coupled with RP-18 silica gel packed glass columns(15 × 230 and 26 × 460 mm, respectively). HPLC system was carried out on a Waters HPLC system (Waters 1525E pumps, Waters 2996 photodiode array detector, Waters fraction collector II) using a analytical semi-preparative or preparative Sunfire C_18_ column (4.6 × 150, 10 × 150, and 19 × 250 mm, respectively).

### Plant Materials

Leaves and stems of *C. lanceolata* and *C. fortunei var. alpina* were collected from Yunnan Province, P. R. China and identified by Dr. Jie Cai, respectively. Two voucher specimen (cai20131002 and cai20140501) was preserved in the State Key Laboratory of Phytochemistry and Plant Resources in West China, Kunming Institute of Botany, Chinese Academy of Sciences.

### Extraction and Isolation of *C. lanceolata* and *C. fortunei var. alpina*

The air-dried and powdered leaves and stems of *C. lanceolata* (19 kg) and *C. fortunei var. alpina* (39 kg) was extracted with MeOH (3 × 50 L, 3 × 100 L, 2 days each) at room temperature, respectively, and the solvent was evaporated in vacuo. The extract was dissolved in 1 % HCl solution (v/v) to pH 2–3, basified with 10 % ammonia solution (v/v) to pH 7–8, and partitioned with EtOAc to afford the crude alkaloids (39 and 198 g).

The alkaloidal extract of *C. lanceolata* (39 g) was subjected to CC over silica gel (400 g) and eluted with a CHCl_3_–MeOH gradient (1:0 to 0:1, v/v) to give four fractions (I-IV) based on TLC analysis. Fraction I (7.5 g) was subjected to C_18_ MPLC with MeOH–H_2_O (20:80 to 100:0, V/V) as the eluent to obtain four fractions (I-1–I-4). I-1 (800 mg) was further separated on a C_18_ MPLC with a gradient of MeOH–H_2_O (20:80 to 40:60, v/v) and then separated on a preparative C_18_ column with a gradient MeOH–H_2_O (30:70 to 40:60, v/v) to afford **6 (**30 mg**)**. I-2 (3 g) was purified on a C_18_ MPLC with a gradient of MeOH–H_2_O (30:20 to 60:40, v/v) to afford the alkaloid **7** (8 mg). **11** (33 mg) was crystallized from I-3 (1 g), and the mother liquid of this fraction was separated on a C_18_ MPLC with a gradient of MeOH–H_2_O (40:60 to 70:30, v/v) to afford the alkaloids **16** (18 mg) and **18** (14 mg). I-4 (2 g) was applied to a C_18_ HPLC with a gradient of MeOH–H_2_O (50:40 to 80:10, v/v) then separated on a preparative C_18_ column with a gradient MeOH–H_2_O (55:45 to 65:35) to obtain **17** (20 mg), **20** (12 mg) and **21** (5.5 mg). Fraction II (15 g) was applied to a C_18_ MPLC with a gradient of MeOH–H_2_O (20:80–100:0, v/v) to obtain four subfractions II-1–II-4. II-1 (5 g) was further applied to a C_18_ MPLC with a gradient of MeOH–H_2_O (10:90 to 70:30, v/v) to give four fractions II-1-1–II-1-4. II-1-1 (0.8 g) was separated on a C_18_ MPLC with a gradient of MeOH–H_2_O (10:90 to 30:70, v/v) and then separated on a preparative C_18_ column with a gradient MeOH–H_2_O (25:75 to 35:65, v/v) to give **1** (8 mg) and **2** (12.5 mg). II-1-3 (2 g) was subjected to a C_18_ MPLC with a gradient of MeOH–H_2_O (30:70 to 60:40, v/v) and then separated on a preparative C_18_ column with a gradient MeOH–H_2_O (48: 52 to 58:42, v/v) to give **12** (55 mg). II-3(4 g) was applied to a C_18_ MPLC with a gradient of MeOH–H_2_O (20:80 to 50:50, v/v) to obtain **27** (9.5 mg), and then separated on a preparative C_18_ column with a gradient MeOH–H_2_O (38:62 to 48:52, v/v) to give **14** (11 mg). II-4 (3.0 g) was subjected to CC over silica gel (30 g) and eluted with a CHCl_3_–MeOH gradient (25:1 to 15:1, v/v) and further purified on a preparative C_18_ column with a gradient MeOH–H_2_O (50:50 to 60:40, v/v) to give **9** (14 mg). III (12 g) was applied to C_18_ MPLC with a gradient of MeOH–H_2_O (20:80 to 60:40, v/v) to obtain four subfractions III-1-III-4. III-1 (4 g) was separated on a C_18_ MPLC with a gradient of MeOH–H_2_O (10:90 to 40:60, v/v) to give **13** (10 mg). III-3 (2.5 g) was separated on a C_18_ MPLC with a gradient of MeOH–H_2_O (30:70 to 60:40, v/v) to give **5** (10 mg) and **22** (22 mg).

The alkaloidal extract of *C. fortunei var. alpina* (198 g) was subjected to CC over silica gel (2.0 kg), eluted with CHCl_3_–MeOH gradient (1:0 to 0:1, v/v) to yield six fractions (I-VI). Fraction II (43 g) was gradually purified C_18_ MPLC with MeOH–H_2_O (30:70 to 100:0, V/V), to afford subfractions II-1–II-6. **6** (200 mg) was crystallized from II-1 (7 g), and the mother liquid of this fraction was separated on a C_18_ MPLC with a gradient of MeOH–H_2_O (30:70 to 50:50, v/v) to afford **7** (5 mg). II-3 (11 g) was subjected to CC over silica gel (120 g) with CHCl_3_–Me_2_CO(20:1 to 5:1, v/v) as the eluent and then further purified on a C_18_ MPLC with a gradient of MeOH–H_2_O (30:70 to 50:50, v/v) to afford **3** (5 mg). II-4 (8 g) was gradually purified on a C_18_ MPLC (MeOH–H_2_O, 40:60 to 60:40, v/v) to afford **20** (98 mg) and then further purified on a preparative C_18_ column with a gradient MeOH–H_2_O (48: 52 to 58:42, v/v) to give **21** (17 mg). II-5 (7 g) was separated by C_18_ MPLC with a gradient of MeOH–H_2_O (50:50 to 70:30, v/v) to give **23** (39 mg). Fraction III (41 g) was separated on a C_18_ MPLC with a gradient of MeOH–H_2_O (20:80 to 100:0, v/v) to afford subfractions (III-1–III-5). Subfraction III-3 (12 g) was gradually separated on a C_18_ MPLC, eluted with MeOH–H_2_O (30:70 to 50:50, v/v) to afford **14** (141 mg). **12** (133 mg) was crystallized from III-5 (13 g), and the mother liquid of this fraction was separated on a C_18_ MPLC with a gradient of MeOH–H_2_O (20:80 to 40:60, v/v) to afford **10** (32 mg). IV (31 g) was separated on a C_18_ MPLC with a gradient of MeOH–H_2_O (10:90 to 100:0, v/v) to yield subfractions IV-1–IV-9. IV-2(8 g) was further purified on a C_18_ MPLC with CH_3_CN–H_2_O (5:95 to 15:85, v/v) as the eluent to give **7** (200 mg). IV-3 (3 g) was subjected to a C_18_ MPLC with MeOH–H_2_O (20:80 to 50:50, v/v), then further purified on a preparative C_18_ column with a gradient MeOH–H_2_O (35:65 to 45:55, v/v) to give **26** (46) and **27** (18 mg). **25** (54 mg) was crystallized from IV-5 (13 g). IV-9 (5 g) was gradually separated on a C_18_ MPLC, eluted with MeOH–H_2_O (35:65 to 55:45, v/v) to afford **28** (100 mg) and **29** (380 mg). V (25 g) was subjected to a C_18_ MPLC with a gradient of MeOH–H_2_O (10:90 to 100:0, v/v) to give five subfractions (V-1–V-5). V-2 (4 g) was separated on a C_18_ MPLC with a gradient of MeOH–H_2_O (10:90 to 30:70, v/v) to afford **4** (600 mg). VI (17 g) was purified on C_18_ MPLC with a gradient of MeOH–H_2_O (10:90 to 100:0, v/v), and VI-3 (3 g) was gradually purified on a C_18_ MPLC (MeOH–H_2_O, 10:90 to 30:70, v/v) and further purified on a preparative C_18_ column with a gradient MeOH–H_2_O (15: 85 to 25:75, v/v) to yield **15** (4 mg), **24** (5 mg) and **19** (18 mg).

Cephalotine A (**1**): white powder; [α]_D_^25^-31.5 (*c* 0.09, MeOH); UV (MeOH) *λ*_max_ (log *ε*) 203 (3.01), 291 (3.91) nm; IR (KBr) *ν*_max_ 3520, 3406, 1631, 1500, 1482, 1342 cm^−1^; ^1^H (400 MHz) and ^13^C NMR (100 MHz) data (acetone-*d*_6_), see Tables 1 and 2; positive HRESIMS *m/z* 318.1336 (calcd for C_17_H_20_NO_5_ [M+H]^+^, 318.1342).

Cephalotine B (**2**): white powder; $[\alpha]_{\rm D}^{25}$ −35.8 (*c* 0.12, MeOH); UV (MeOH) *λ*_max_ (log *ε*) 204 (2.95), 291 (3.81) nm; IR (KBr) *ν*_max_ 3450, 3430, 1631, 1484, 1342 cm^−1^; ^1^H (400 MHz) and ^13^C NMR (100 MHz) data (acetone-*d*_6_), see Tables 1 and 2; positive HRESIMS *m/z* 318.1335 (calcd for C_17_H_20_NO_5_ [M+H]^+^, 318.1342).

Cephalotine C (**3**): brown oil; $[\alpha ]_{\text{D}}^{25}$ +9.0 (*c* 0.13, MeOH); UV (MeOH) *λ*_max_ (log *ε*) 237(3.67), 280 (3.80) nm; IR (KBr) *ν*_max_ 3437, 2954, 1752, 1735, 1654, 1223 cm^−1^; ^1^H (600 MHz) and ^13^C NMR (150 MHz) data (DMSO-*d*_*6*_), see Tables 1 and 2; positive HRESIMS *m/z* 330.1337 (calcd for C_18_H_20_NO_5_ [M+H]^+^, 330.1336).

Cephalotine D (**4**): colorless powder; $[\alpha ]_{\text{D}}^{25}$ +138.0 (*c* 0.41, MeOH); UV (MeOH) *λ*_max_ (log *ε*) 240 (3.84), 279 (3.89) nm; IR (KBr) *ν*_max_ 3437, 2922, 1659, 1590, 1130 cm^−1^; ^1^H (400 MHz) and ^13^C NMR (100 MHz) data (acetone-*d*_6_), see Tables 1 and 2; positive HRESIMS *m/z* 374.1604 (calcd for C_20_H_24_NO_6_ [M+H]^+^, 374.1598).

Cephalotine E (**5**): white powder; $[\alpha ]_{\text{D}}^{25}$ +46.3 (*c* 0.10, MeOH); UV (MeOH) *λ*_max_ (log *ε*) 204 (3.66), 243 (2.73), 288 (2.67)nm; IR (KBr) *ν*_max_ 3419, 2934, 1623, 1507, 1490 cm^−1^; ^1^H (400 MHz) and ^13^C NMR (100 MHz) data (acetone-*d*_*6*_), see Tables 1 and 2; positive HRESIMS *m/z* 330.1717 (calcd for C_20_H_24_NO_4_ [M+H]^+^, 330.1705).

### Cytotoxicity Assay

Three human cancer cell lines, HeLa, SGC-7901 gastric cancer, and A-549 lung cancer, were used in the cytotoxicity assay. All the cells were cultured in RPMI-1640 or DMEM media (Hyclone, USA), supplemented with 10 % fetal bovine serum (Hyclone, USA) in 5 % CO_2_ at 37 °C. The cytotoxicity assay was performed according to the MTT (3-(4,5-dimethylthiazol-2-yl)-2,5-diphenyl tetrazolium bromide) method in 96-well microplates. Briefly, 100 *µ*L adherent cells were seeded into each well of 96-well cell culture plates and allowed to adhere for 12 h before addition of the test compound/drug. Meanwhile suspended cells were seeded with initial density of 1 × 10^5^ cells/mL just before addition of the test compound/drug. Each tumor cell line was exposed to the test compound at concentrations of 0.06, 0.32, 1.60, 8.0, and 40 *μ*M for 48 h. Each of these tests was conducted in triplicate, with cisplatin (sigma, USA) as the positive control. After the end of the treatment period, cell viability was measured and cell growth curve was plotted.

## Electronic Supplementary Material

Below is the link to the electronic supplementary material.

Supplementary material 1 (DOCX 6135 kb)
