# Supplementary material for: Five New Alkaloids from Cephalotaxus lanceolata and C. fortunei var. alpina
Source: Nat Prod Bioprospect. 2016 Mar 26;6(3):149–54. doi: 10.1007/s13659-016-0093-7 (PMC5385657; doi:10.1007/s13659-016-0093-7)
Supplement: Supplementary file 1 — Supplementary material 1 (DOCX 6135 kb) [file 13659_2016_93_MOESM1_ESM.docx]

**Electronic Supporting Information**

**Five New Alkaloids from *Cephalotaxus lanceolata* and *C. fortunei var. alpina***

Ling Ni^†‡^, Xiu-Hong Zhong^†‡^, Jie Cai^§^, Mei-Fen Bao^†^, Bing-Jie Zhang^†‡^, Jing Wu^†^, and Xiang-Hai Cai^*,†^

Ling Ni and Xiu-Hong Zhong have contributed equally to this work.

^†^State Key Laboratory of Phytochemistry and Plant Resources in West China, Kunming Institute of Botany, Chinese Academy of Scienes, Kunming 650201, People’s Republic of China

^‡^University of Chinese Academy of Sciences, Beijing 100039, People’s Republic of China

^§^Germplasm Bank of Wild Species in Southwest China, Kunming Institute of Botany, Chinese Academy of Sciences, Kunming 650201, People’s Republic of China.

**Contents**

**Fig S1**. ^1^H NMR spectrum of **1** in acetone-*d*_6_.

**Fig S2**. ^13^C and DEPT NMR spectrum of **1** in acetone-*d*_6_.

**Fig S3**. HSQC spectrum of **1** in acetone-*d*_6_.

**Fig S4**. HMBC spectrum of **1** in acetone-*d*_6_.

**Fig S5**. ^1^H-^1^H COSY spectrum of **1** in acetone-*d*_6_

**Fig S6**. ROESY spectrum of **1** in acetone-*d*_6_.

**Fig S7**. ^1^H NMR spectrum of **2** in acetone-*d*_6_.

**Fig S8**. ^13^C and DEPT spectrum of **2** in acetone-*d*_6_.

**Fig S9**. HSQC spectrum of **2** in acetone-*d*_6_.

**Fig S10**. HMBC spectrum of **2** in acetone-*d*_6_.

**Fig S11**. ^1^H-^1^H COSY spectrum of **2** in acetone-*d*_6_

**Fig S12**. ROESY spectrum of **2** in acetone-*d*_6_.

**Fig S13**. ^1^H NMR spectrum of **3** in DMSO-*d_6_*.

**Fig S14**. ^13^C and DEPT spectrum of **3** in DMSO-*d_6_*.

**Fig S15**. HSQC spectrum of **3** in DMSO-*d_6_*.

**Fig S16**. HMBC spectrum of **3** in DMSO-*d_6_*.

**Fig S17**. ROESY spectrum of **3** in DMSO-*d_6_*.

**Fig S18**. ^1^H NMR spectrum of **4** in acetone-*d*_6_.

**Fig S19**. ^13^C and DEPT spectrum of **4** in acetone-*d*_6_.

**Fig S20**. HSQC spectrum of **4** in acetone-*d*_6_.

**Fig S21**. HMBC spectrum of **4** in acetone-*d*_6_.

**Fig S22**. ROESY spectrum of **4** in acetone-*d*_6_.

**Fig S23**. ^1^H NMR spectrum of **5** in acetone -*d_6_*.

**Fig S24**. ^13^C and DEPT spectrum of **5** in acetone -*d_6_*.

**Fig S25**. HSQC spectrum of **5** in acetone-*d*_6_.

**Fig S26**. HMBC spectrum of **5** in acetone-*d*_6_.

**Fig S27**. ROESY spectrum of **5** in acetone-*d*_6_.


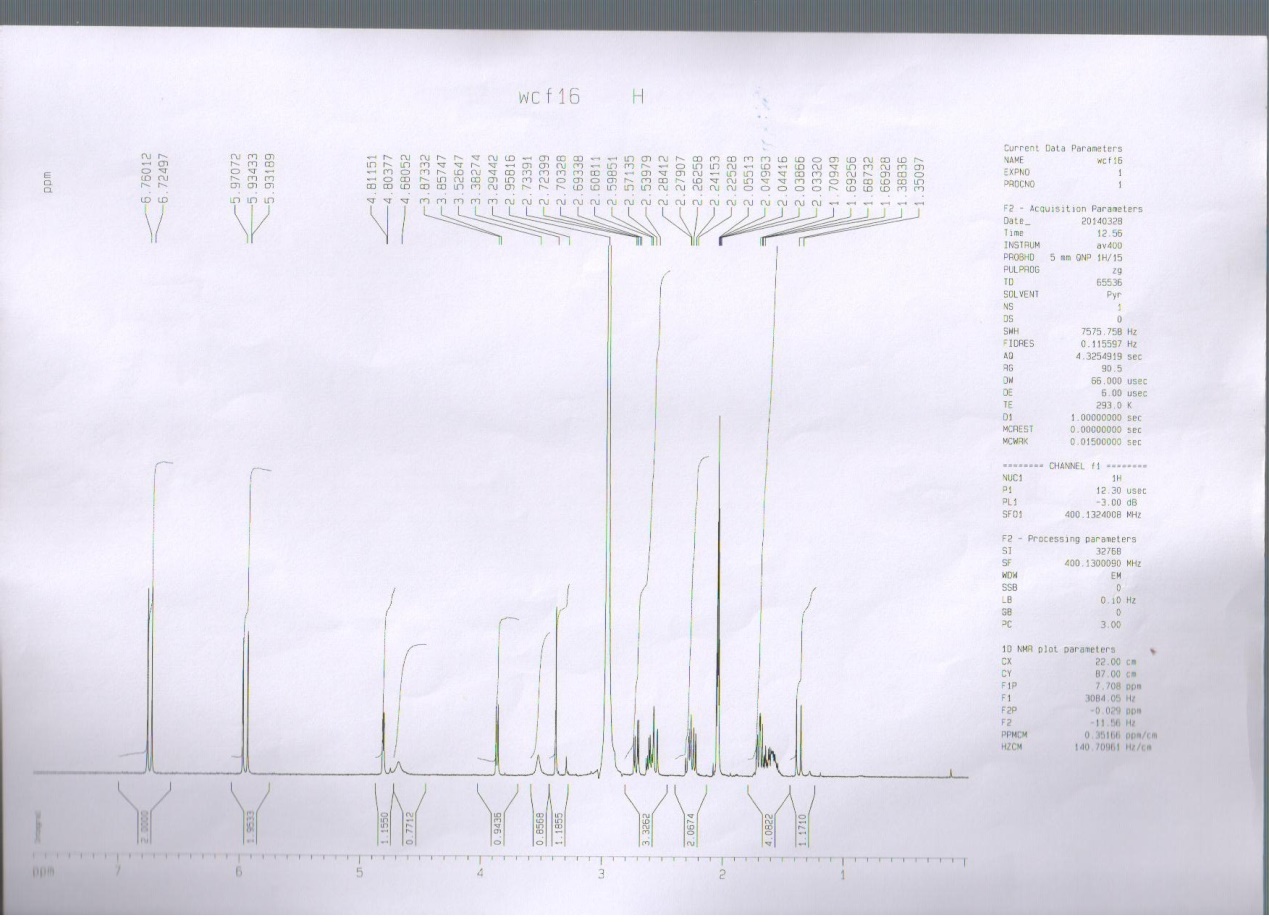


**Fig S1**. ^1^H NMR spectrum of **1** in acetone-*d*_6_.


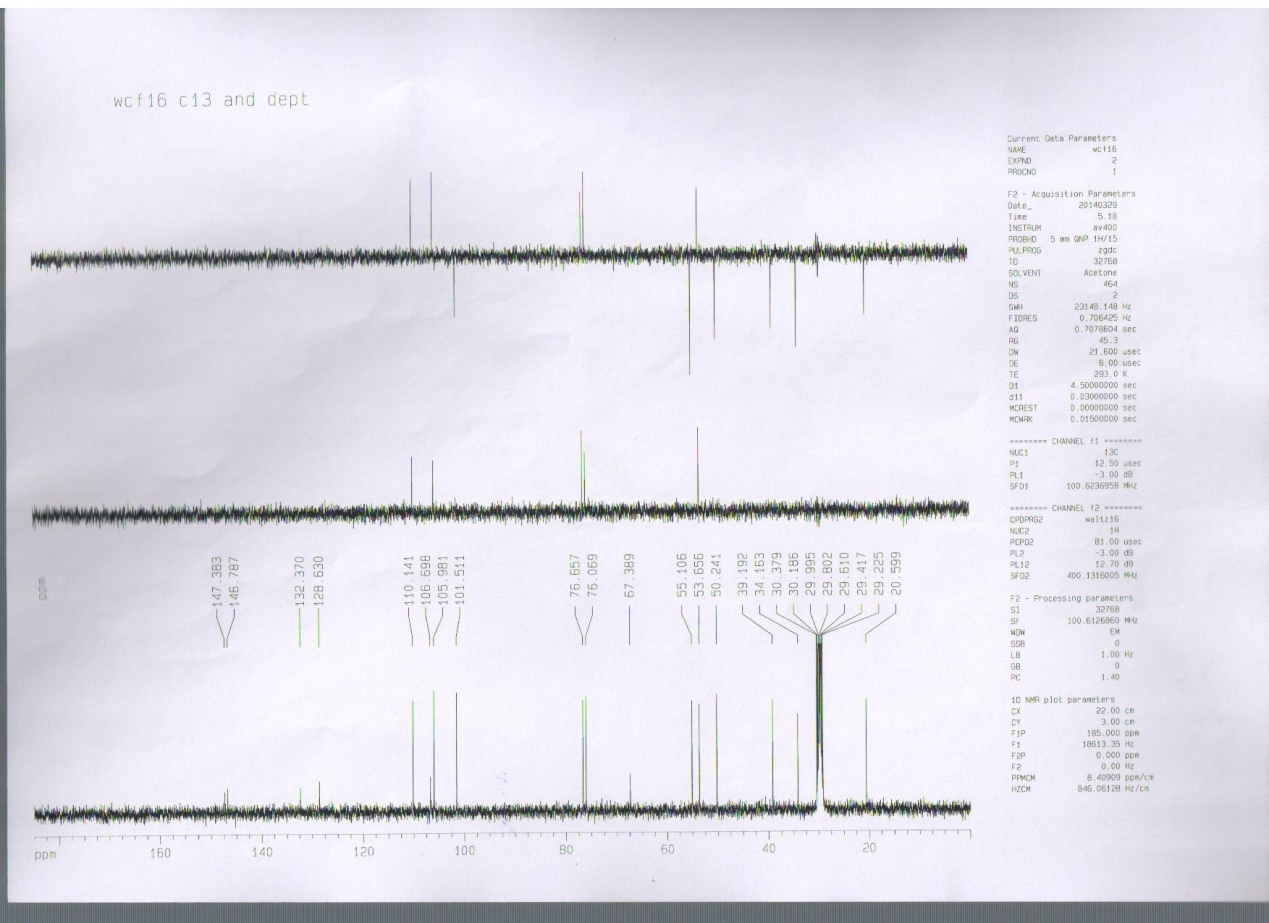


**Fig S2**. ^13^C and DEPT NMR spectrum of **1** in acetone-*d*_6_.


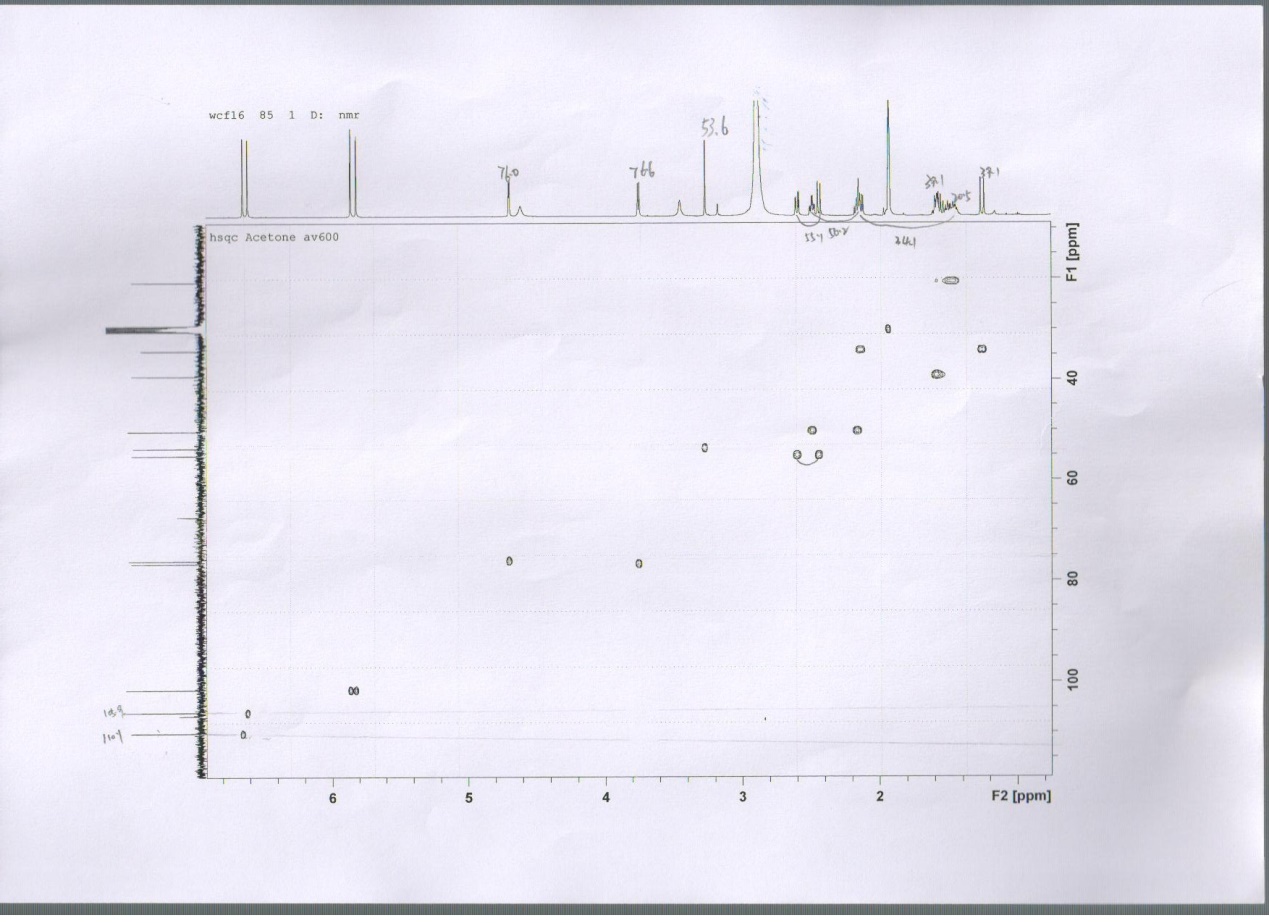


**Fig S3**. HSQC spectrum of **1** in acetone-*d*_6_.


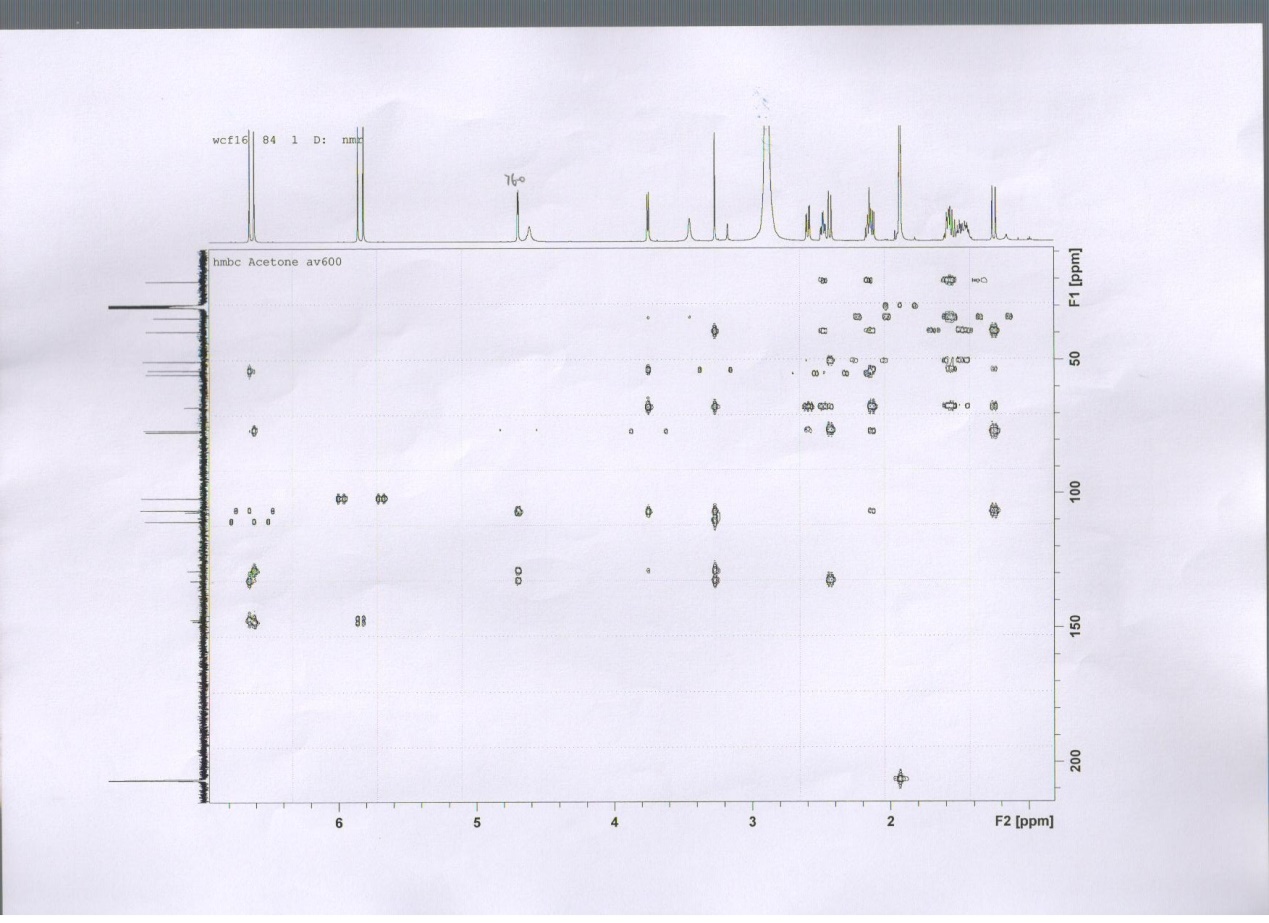


**Fig S4**. HMBC spectrum of **1** in acetone-*d*_6_.


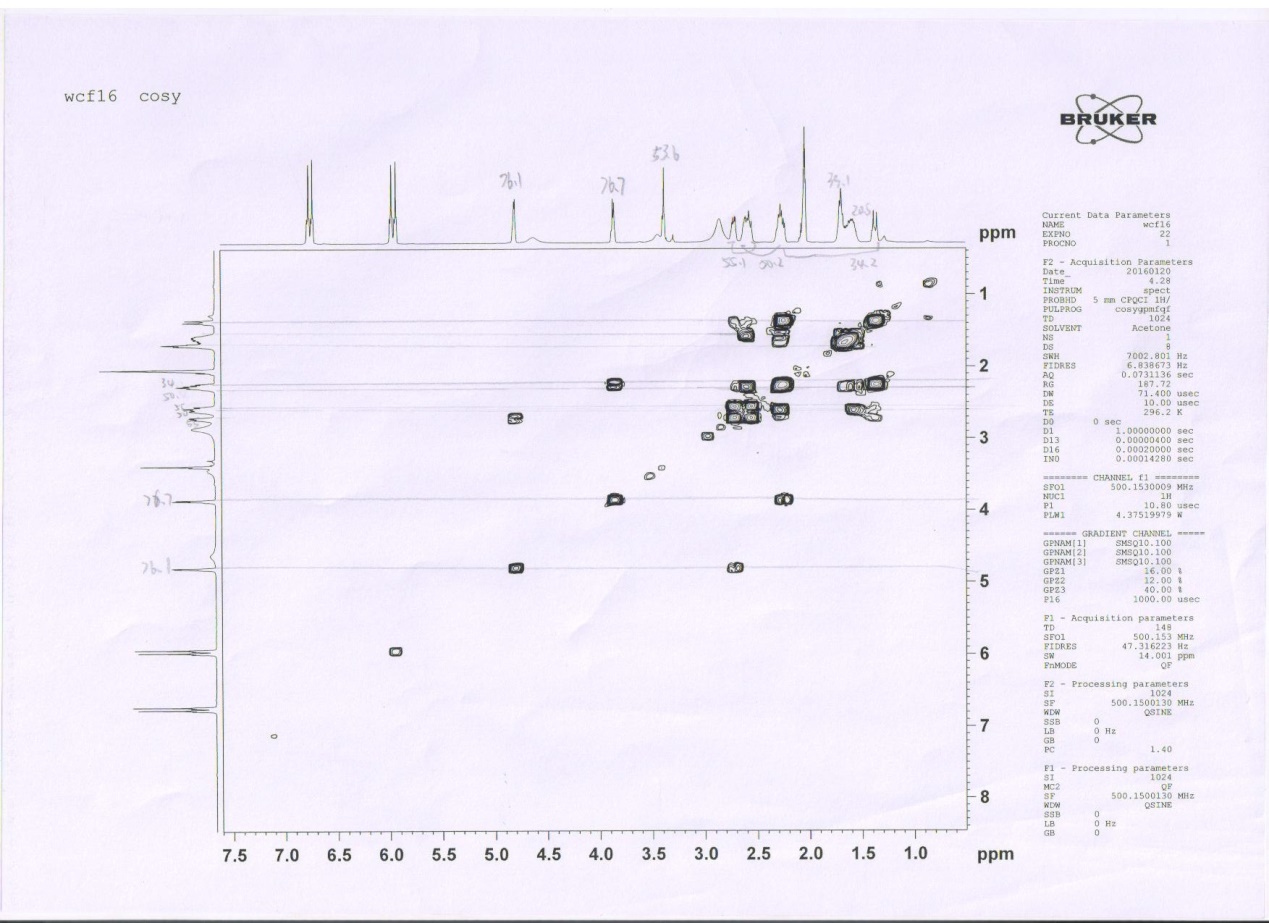


**Fig S5**. ^1^H-^1^H COSY spectrum of **1** in acetone-*d*_6_


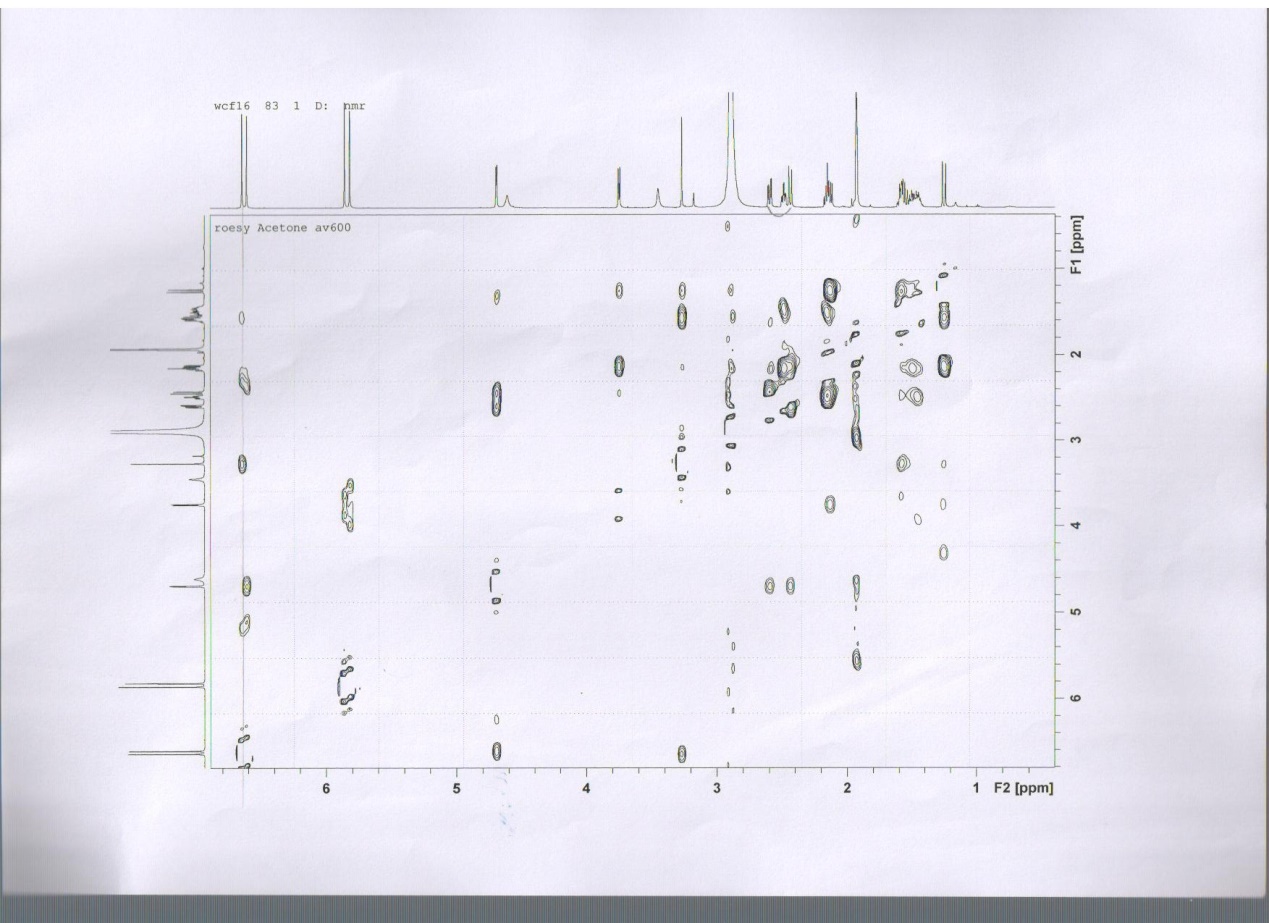


**Fig S6**. ROESY spectrum of **1** in acetone-*d*_6_.


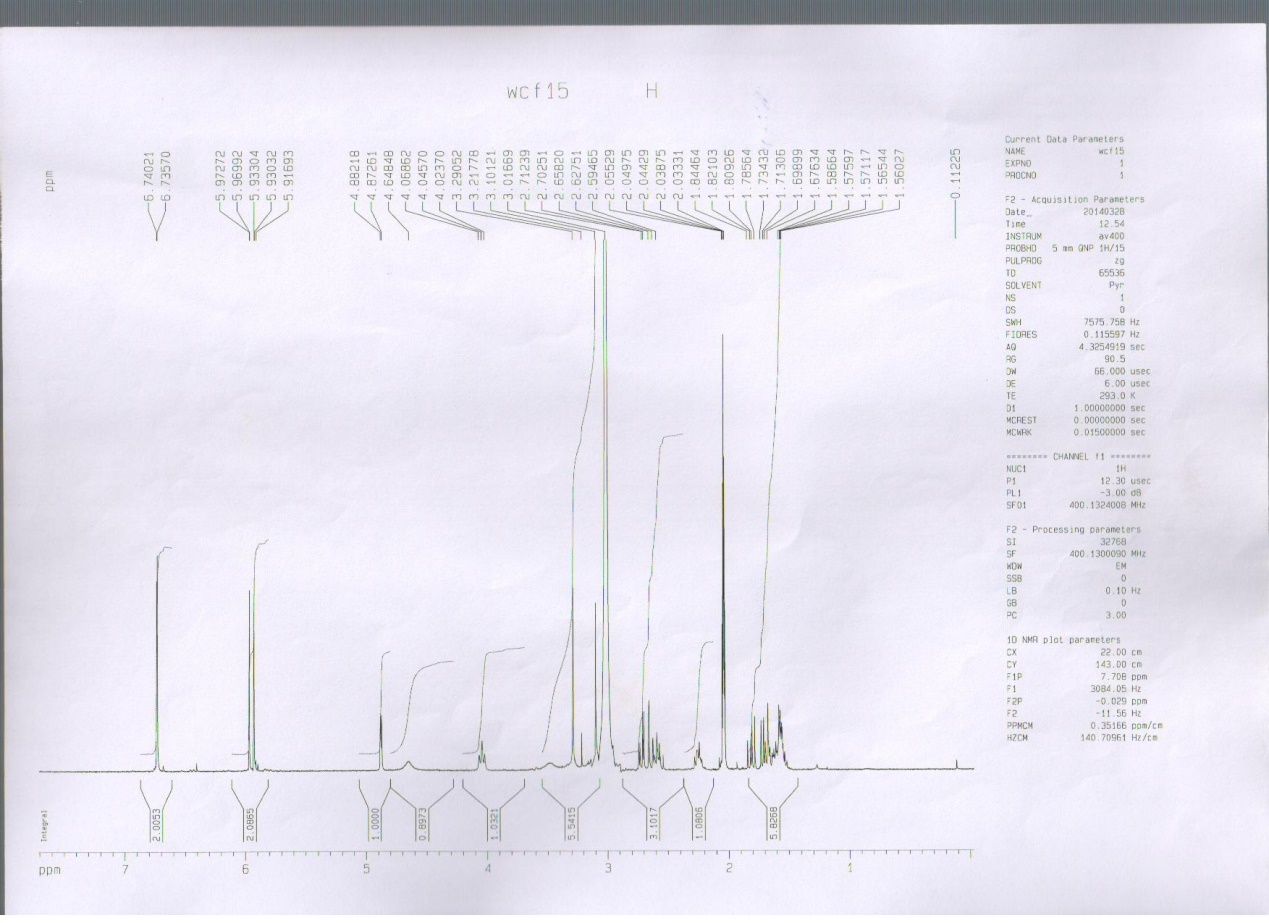


**Fig S7**. ^1^H NMR spectrum of **2** in acetone-*d*_6_.


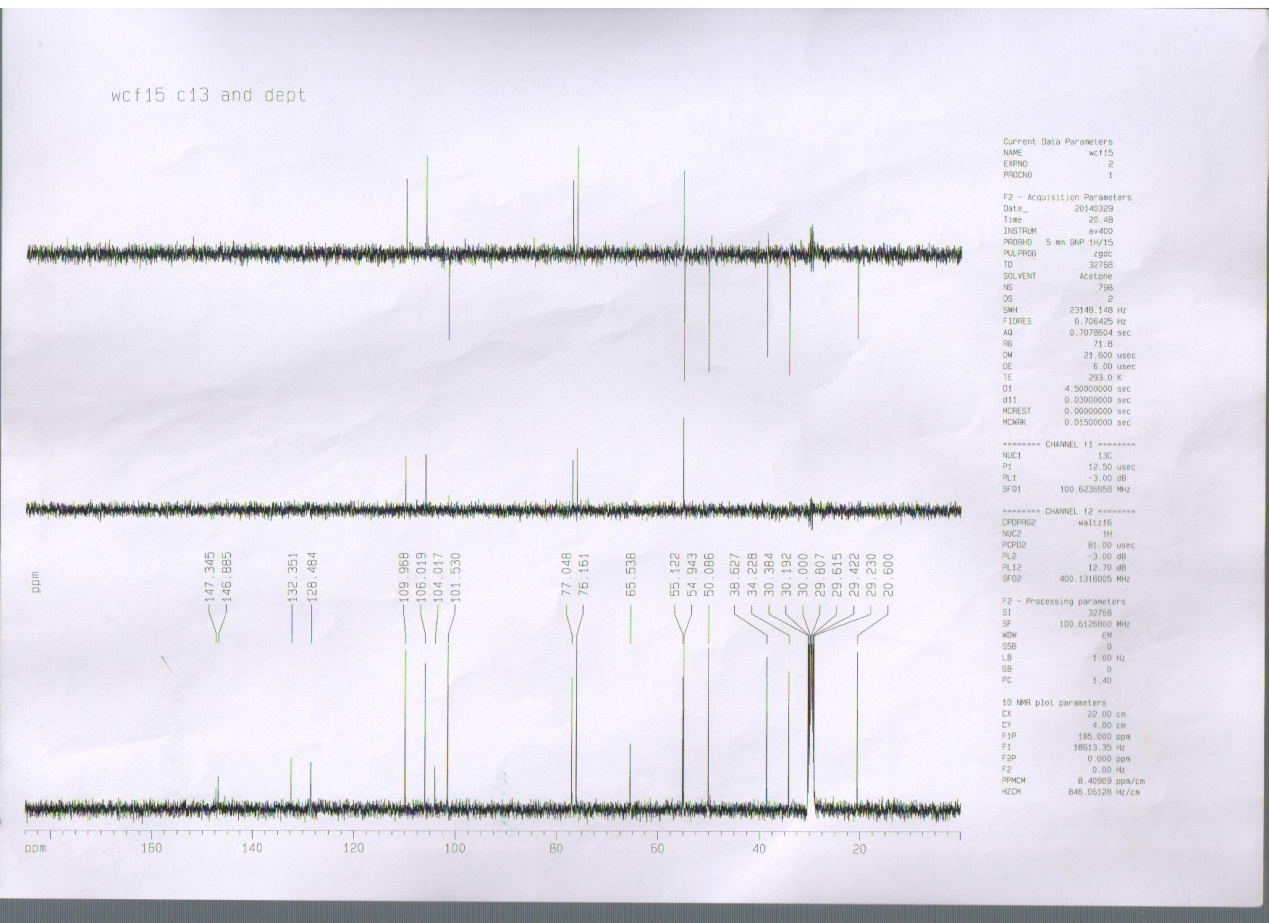


**Fig S8**. ^13^C and DEPT spectrum of **2** in acetone-*d*_6_.


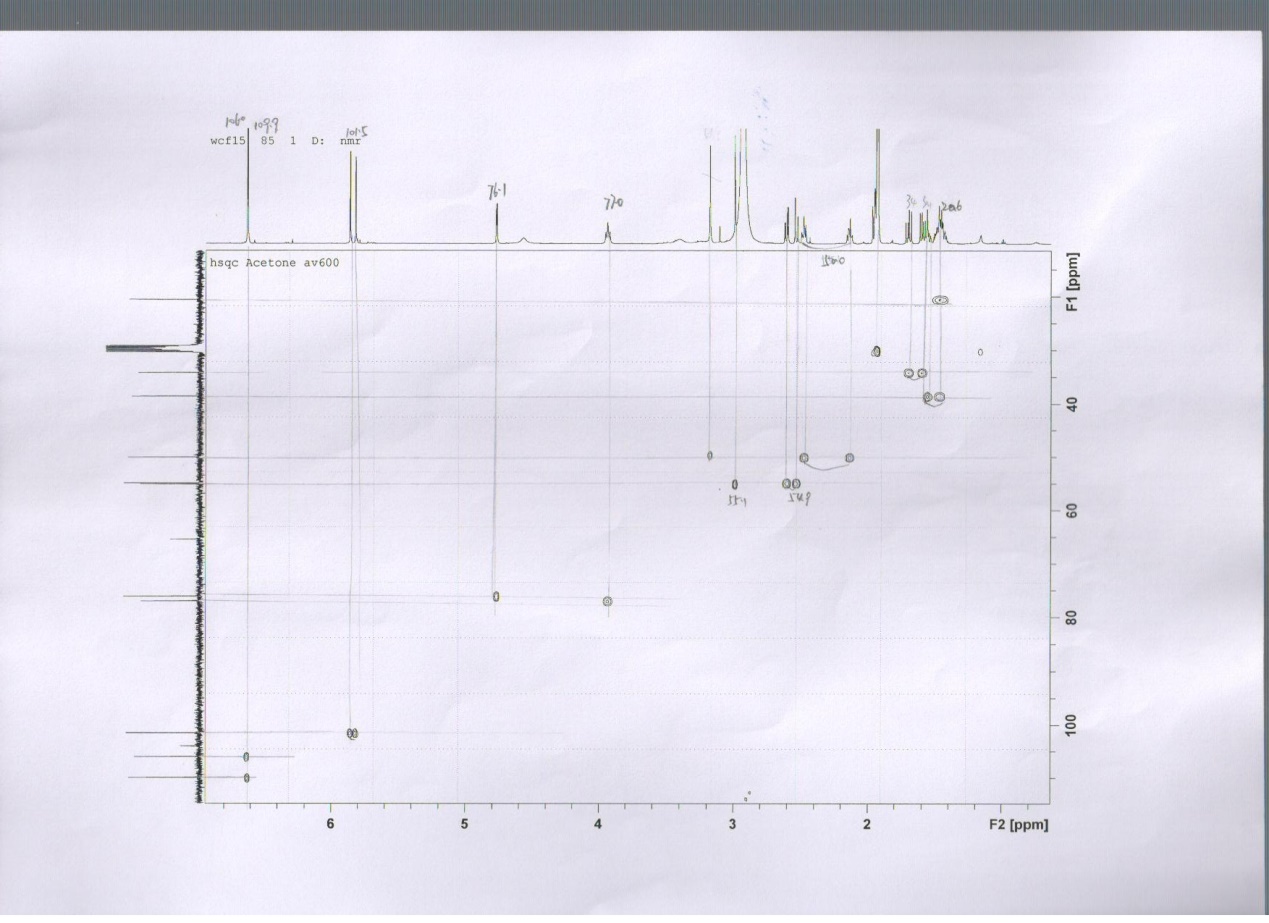


**Fig S9**. HSQC spectrum of **2** in acetone-*d*_6_.


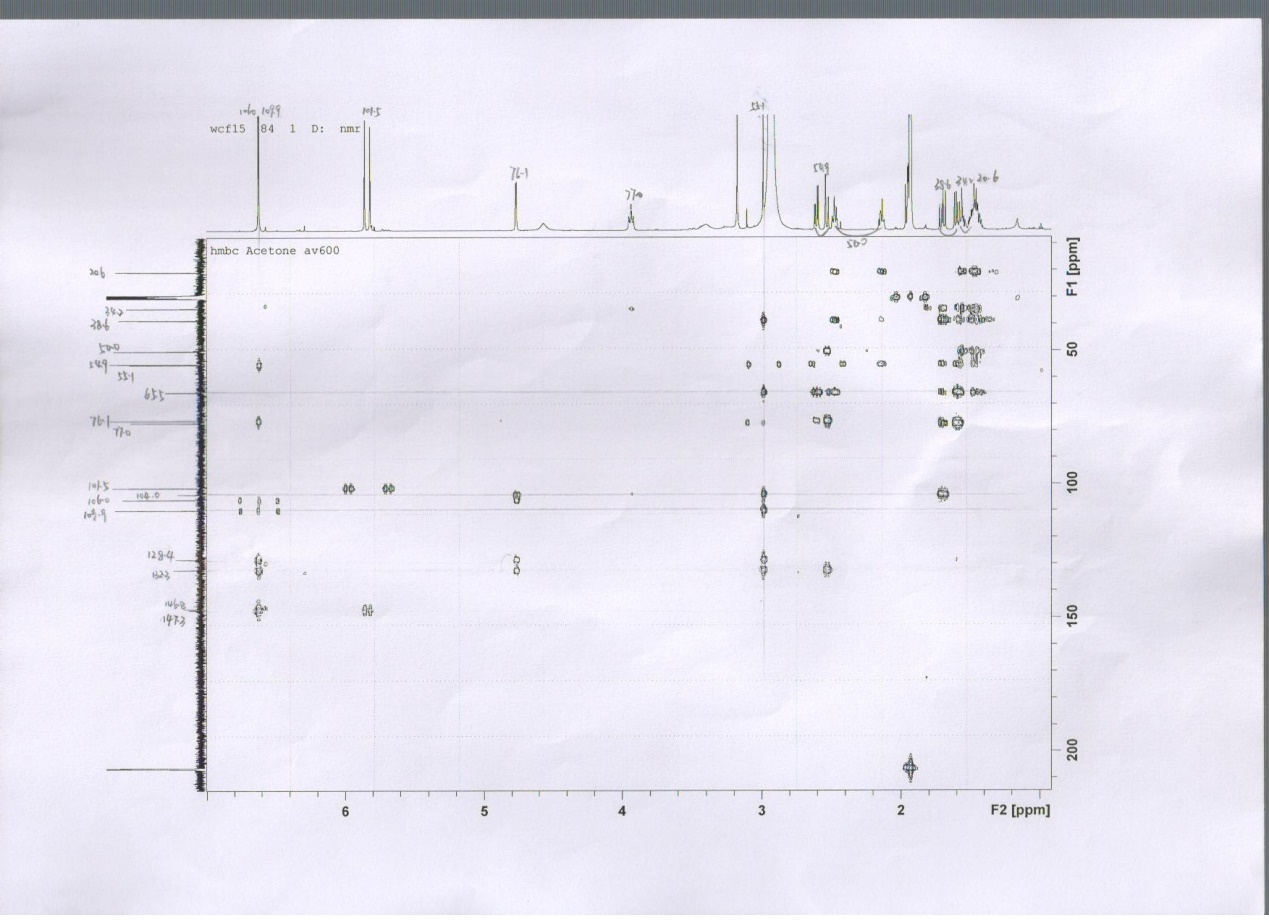


**Fig S10**. HMBC spectrum of **2** in acetone-*d*_6_.


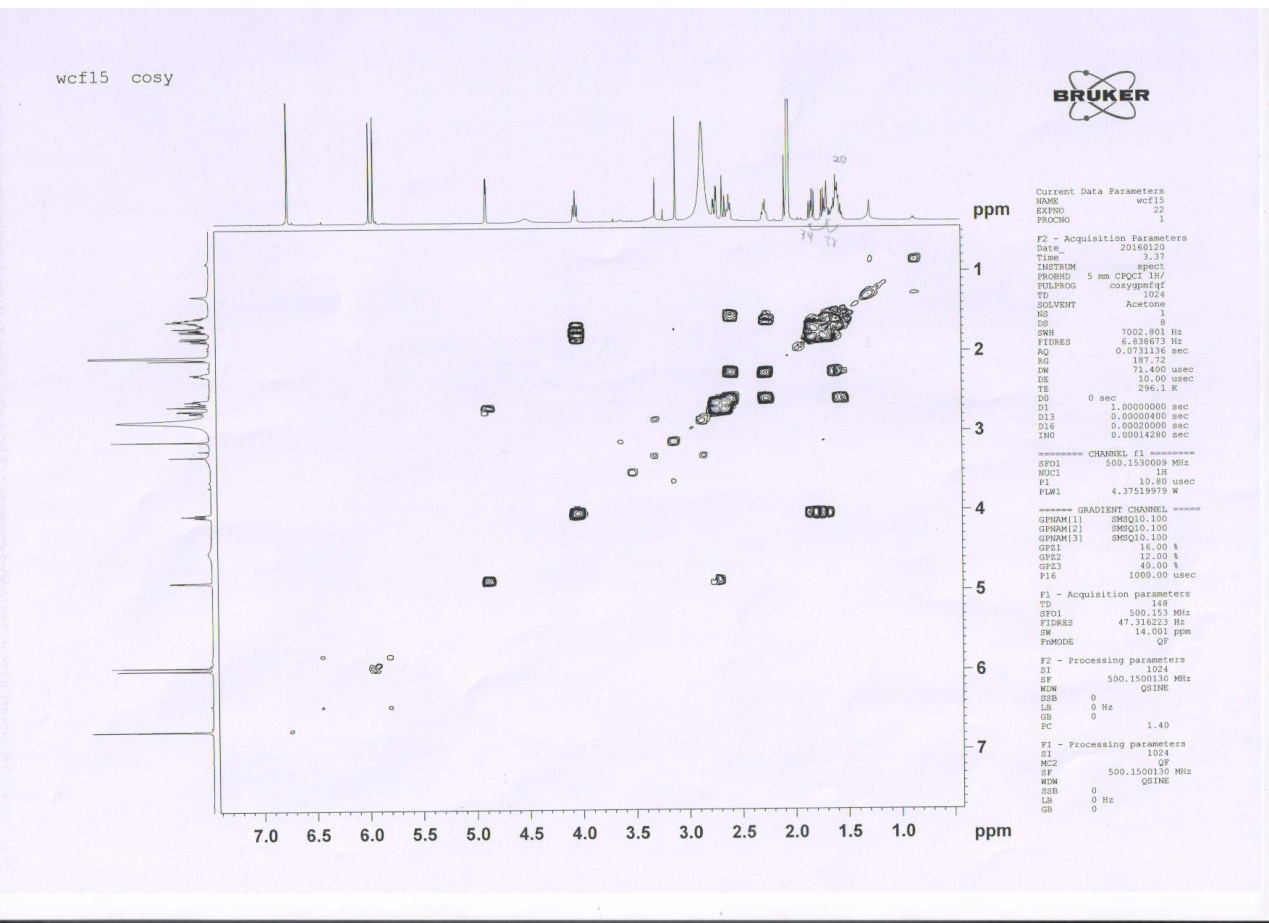


**Fig S11**. ^1^H-^1^H COSY spectrum of **2** in acetone-*d*_6_


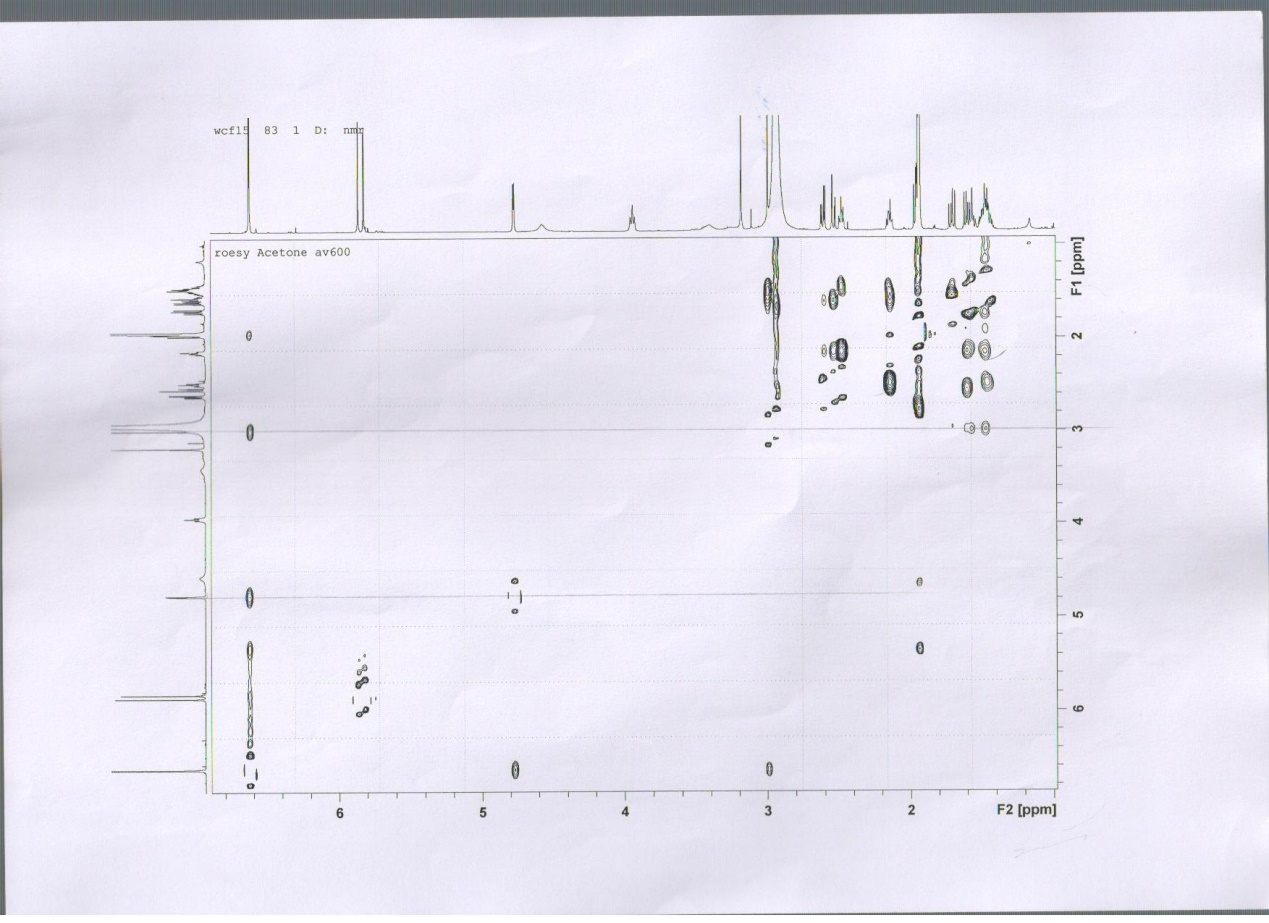


**Fig S12**. ROESY spectrum of **2** in acetone-*d*_6_.


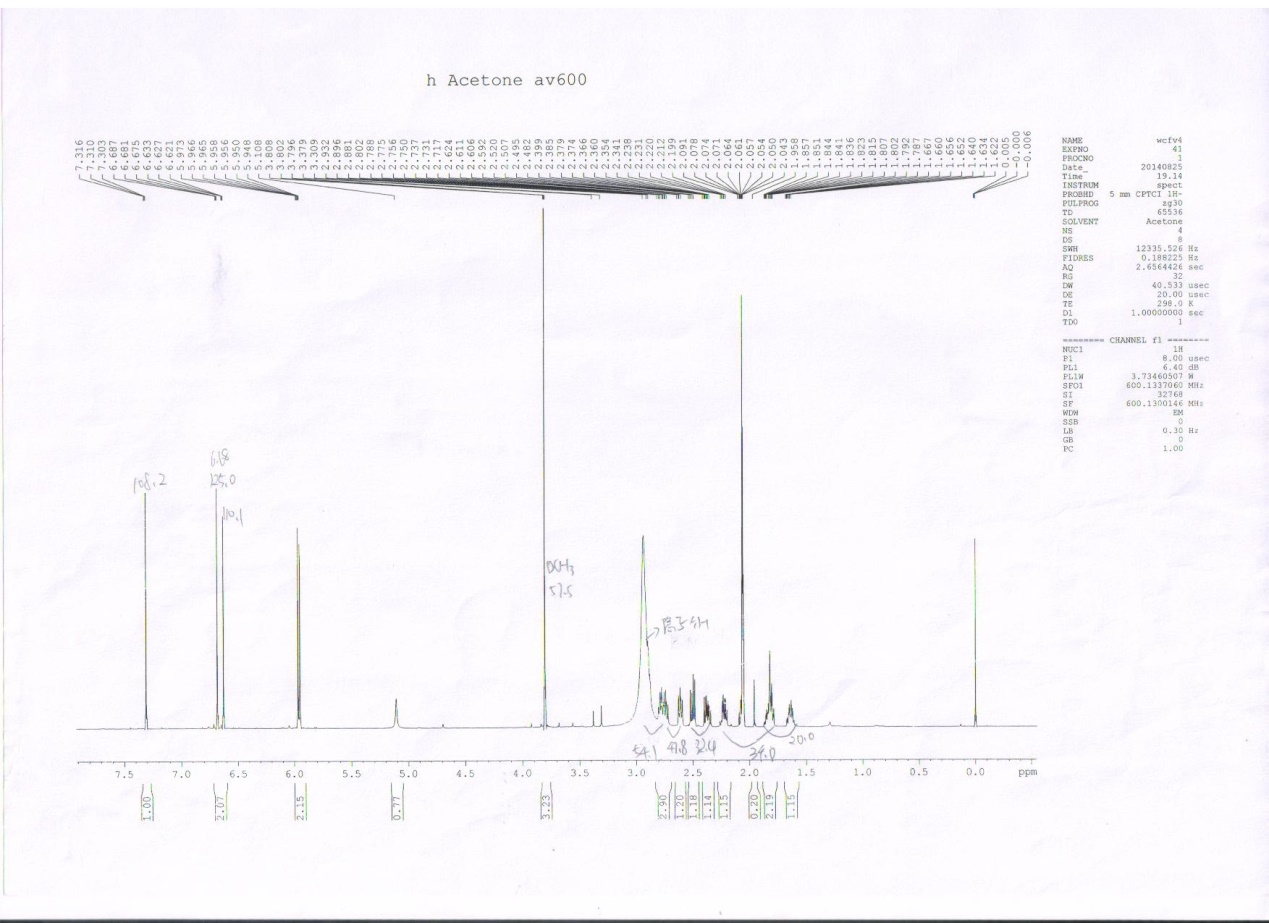


**Fig S13**. ^1^H NMR spectrum of **3** in DMSO-*d_6_*.


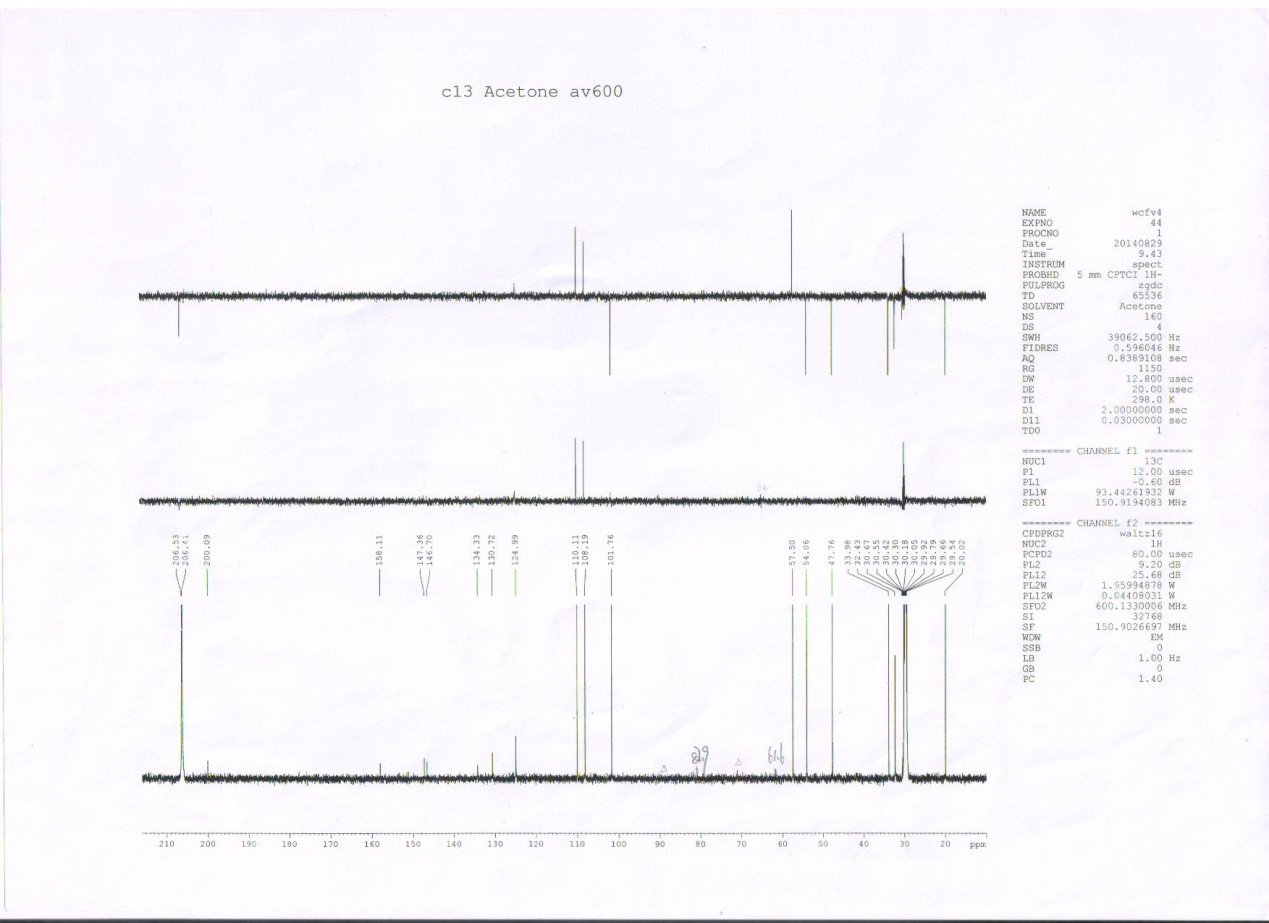


**Fig S14**. ^13^C and DEPT spectrum of **3** in DMSO-*d_6_*.


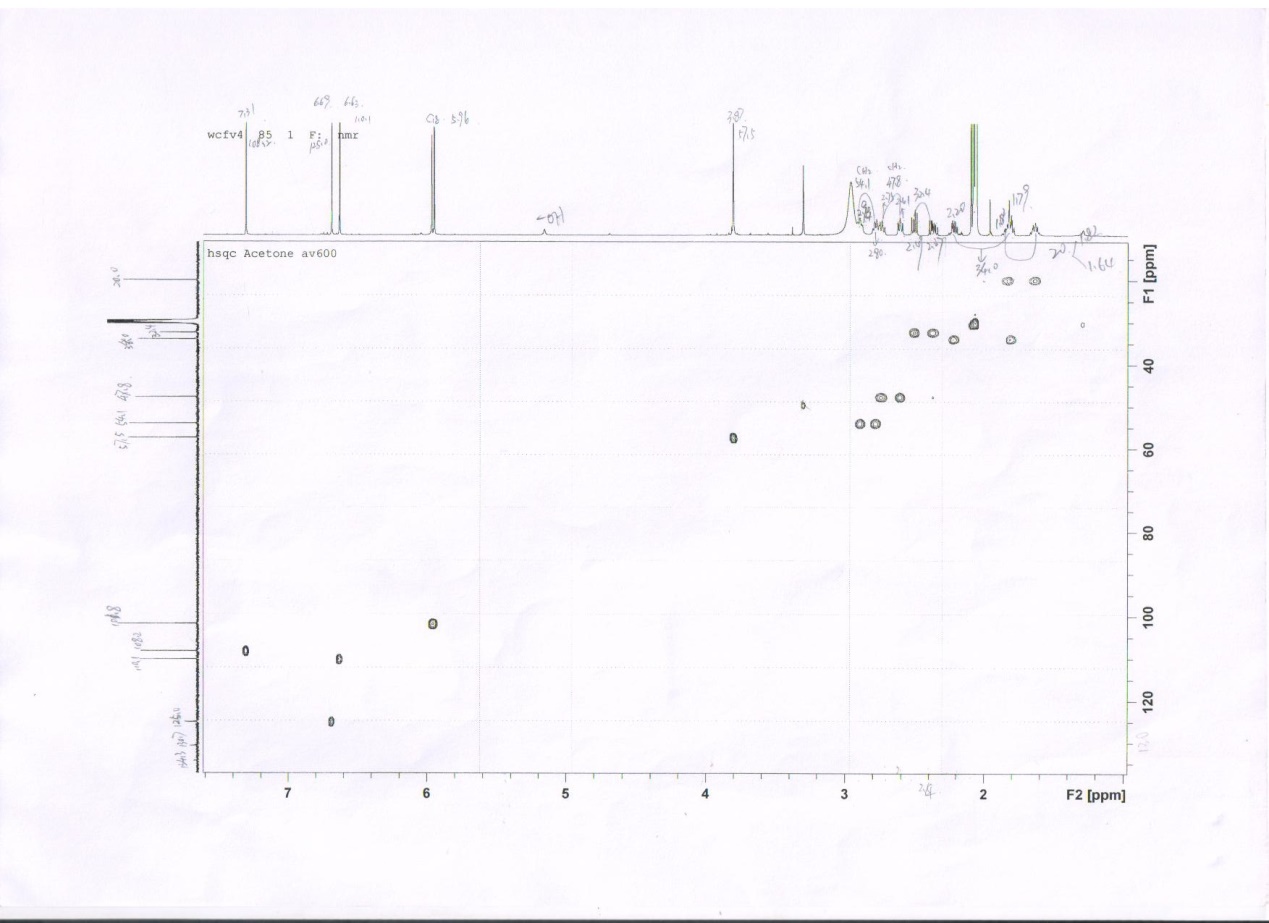


**Fig S15**. HSQC spectrum of **3** in acetone-*d*_6_.


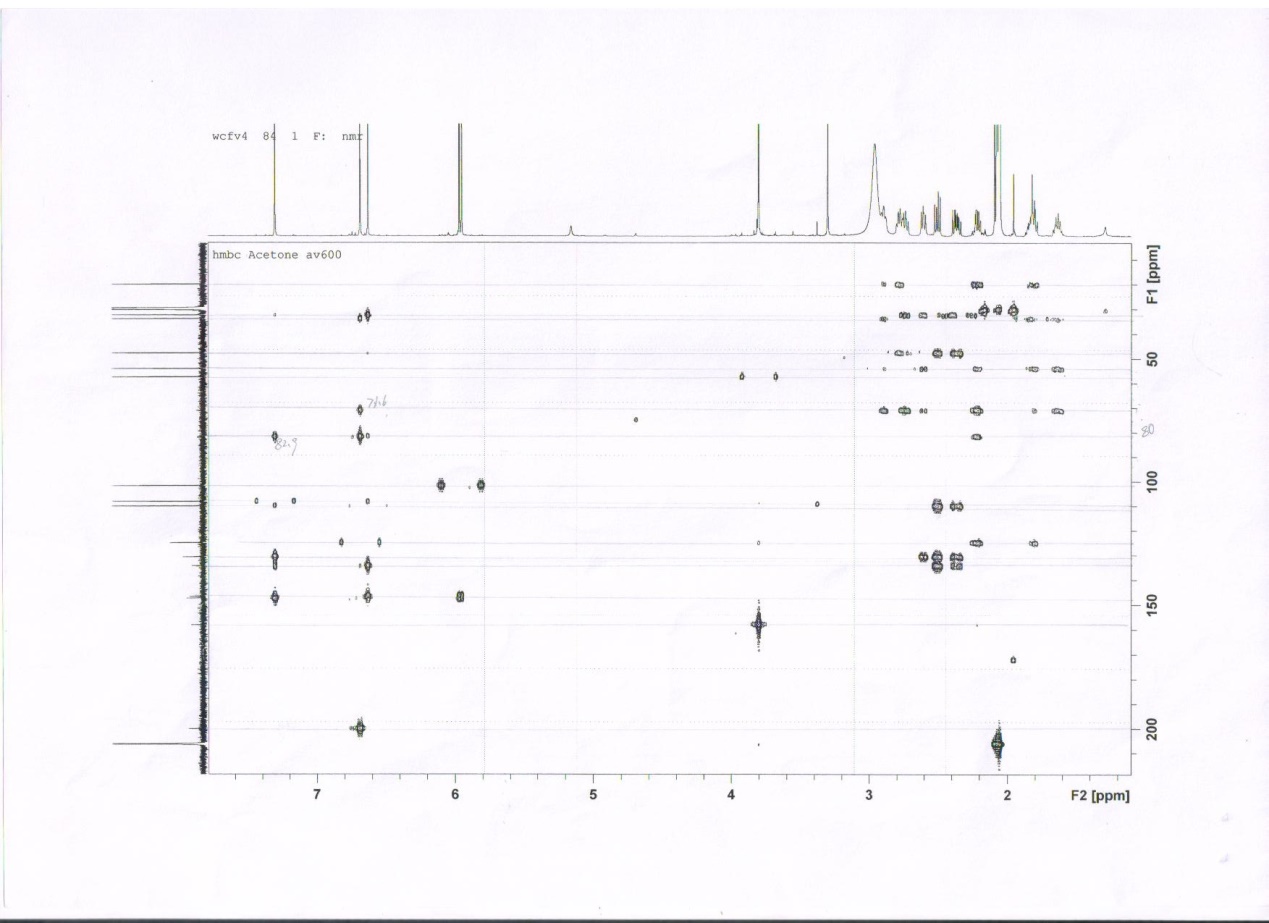


**Fig S16**. HMBC spectrum of **3** in DMSO-*d_6_*.


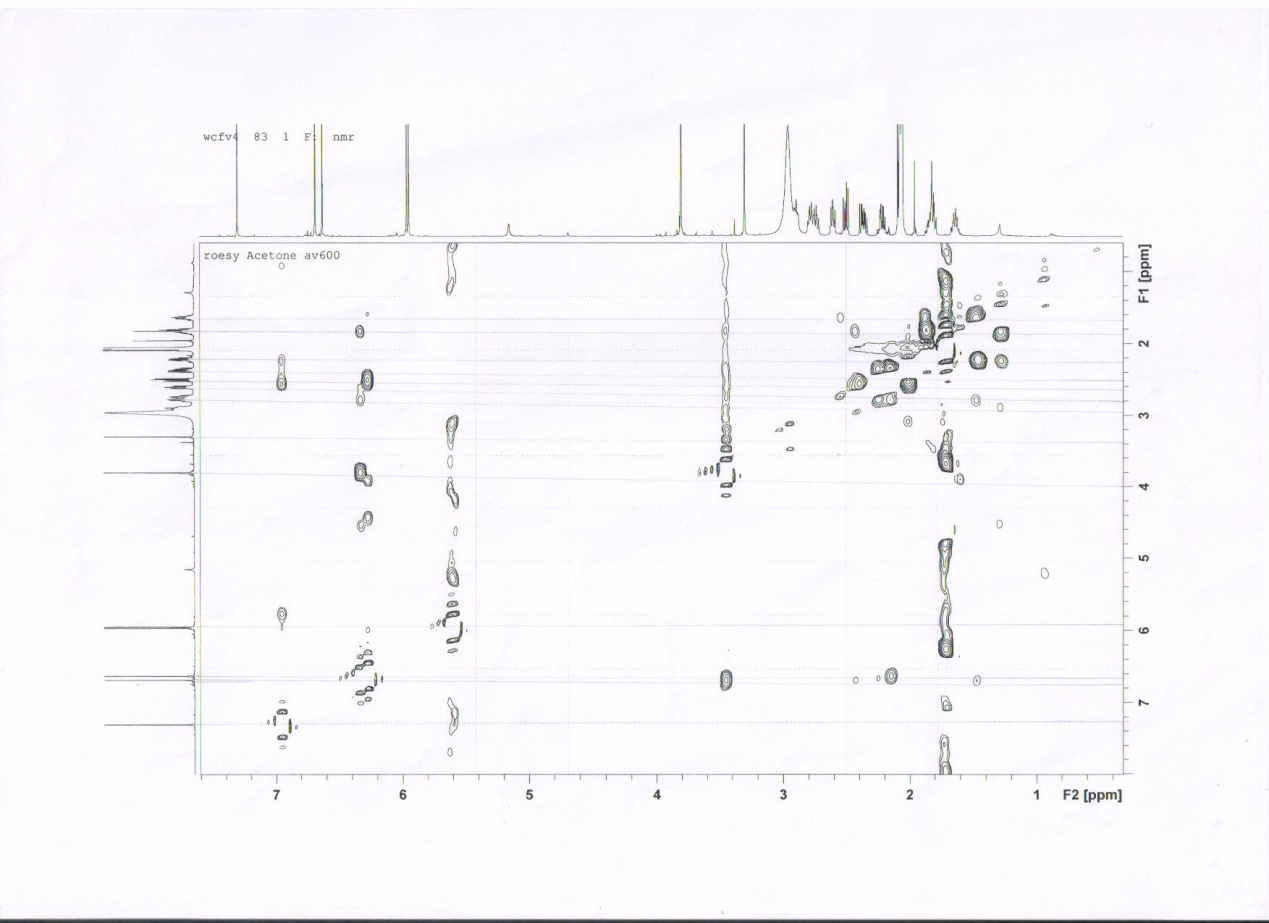


**Fig S17**. ROESY spectrum of **3** in DMSO-*d_6_*.


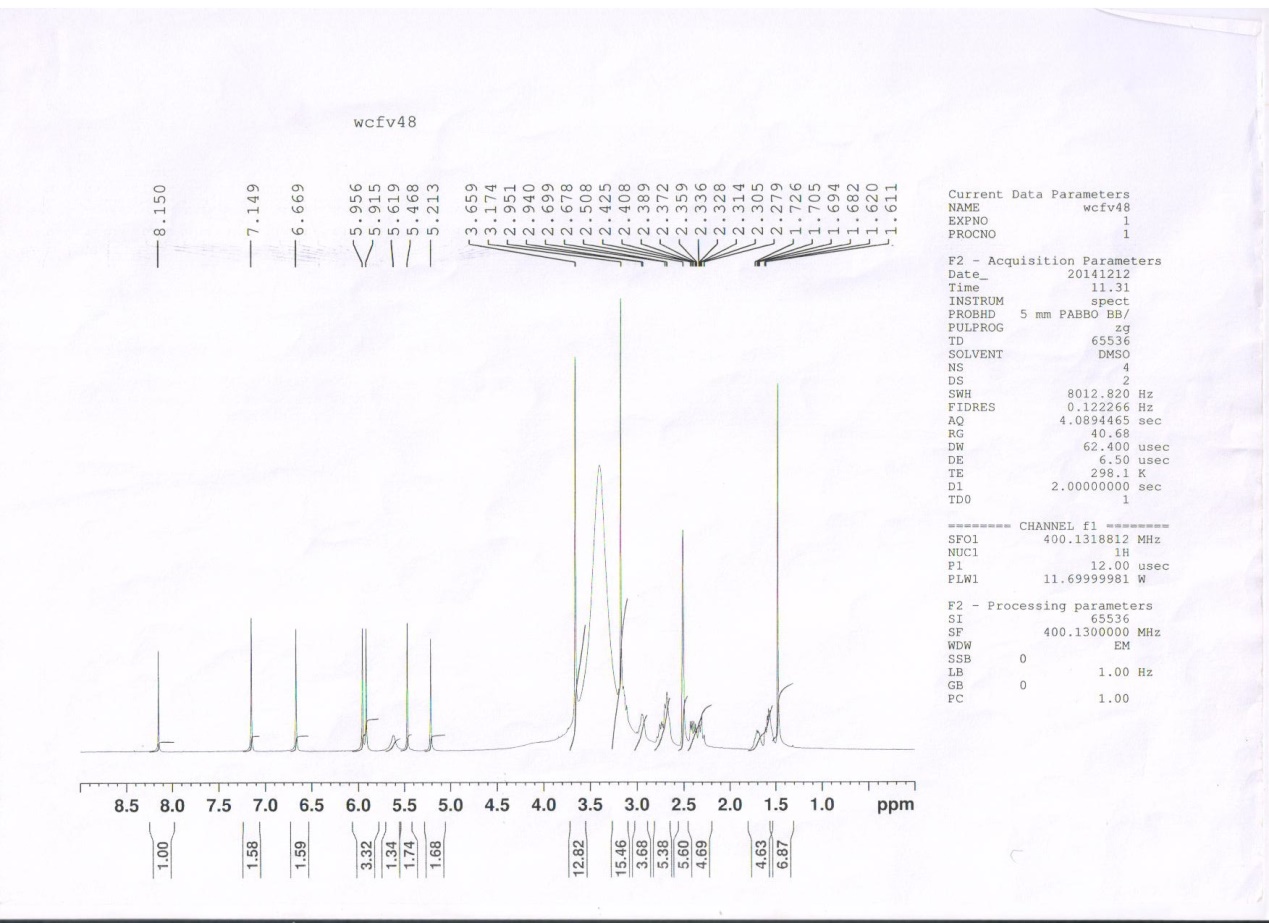


**Fig S18**. ^1^H NMR spectrum of **4** in acetone-*d*_6_.


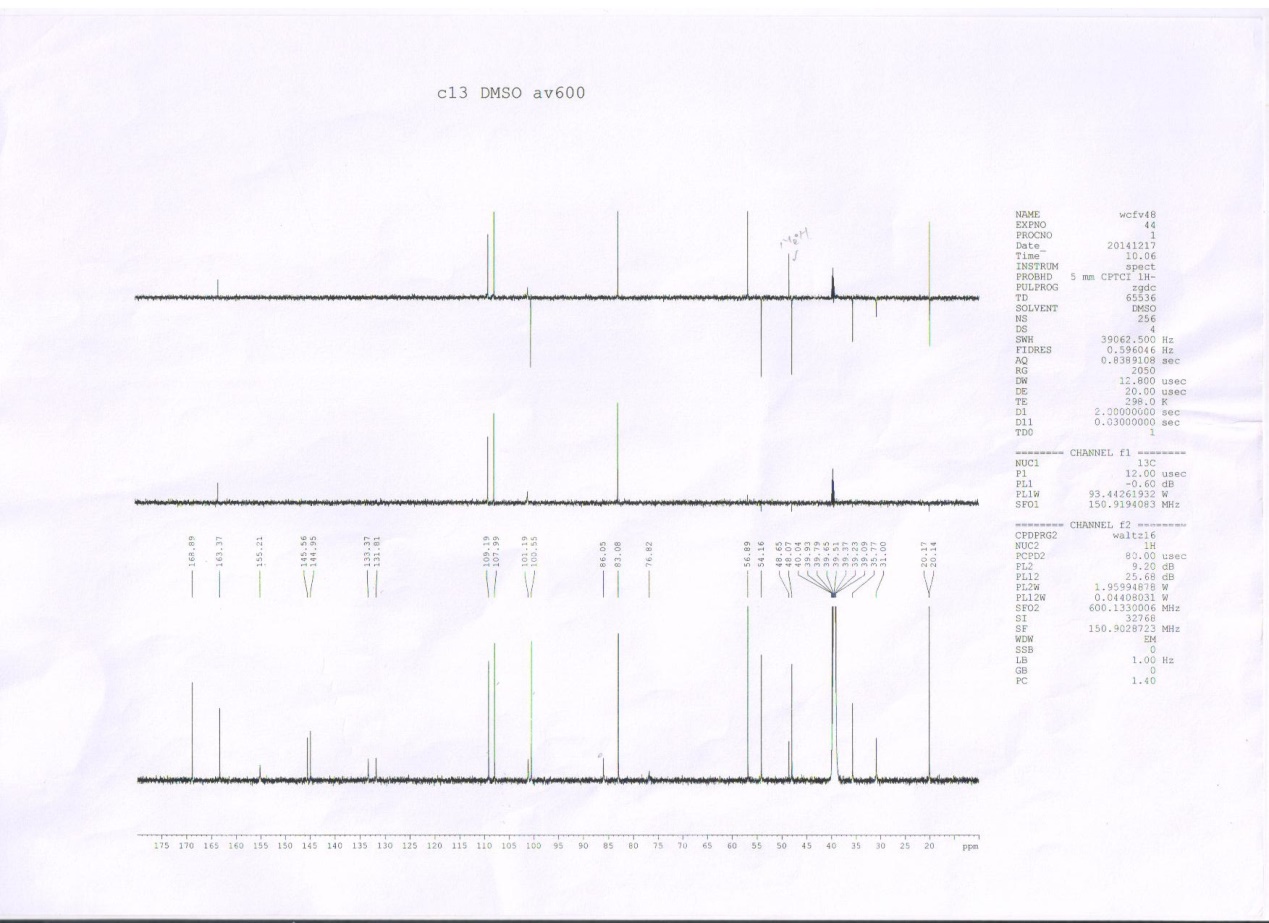


**Fig S19**. ^13^C and DEPT spectrum of **4** in acetone-*d*_6_.


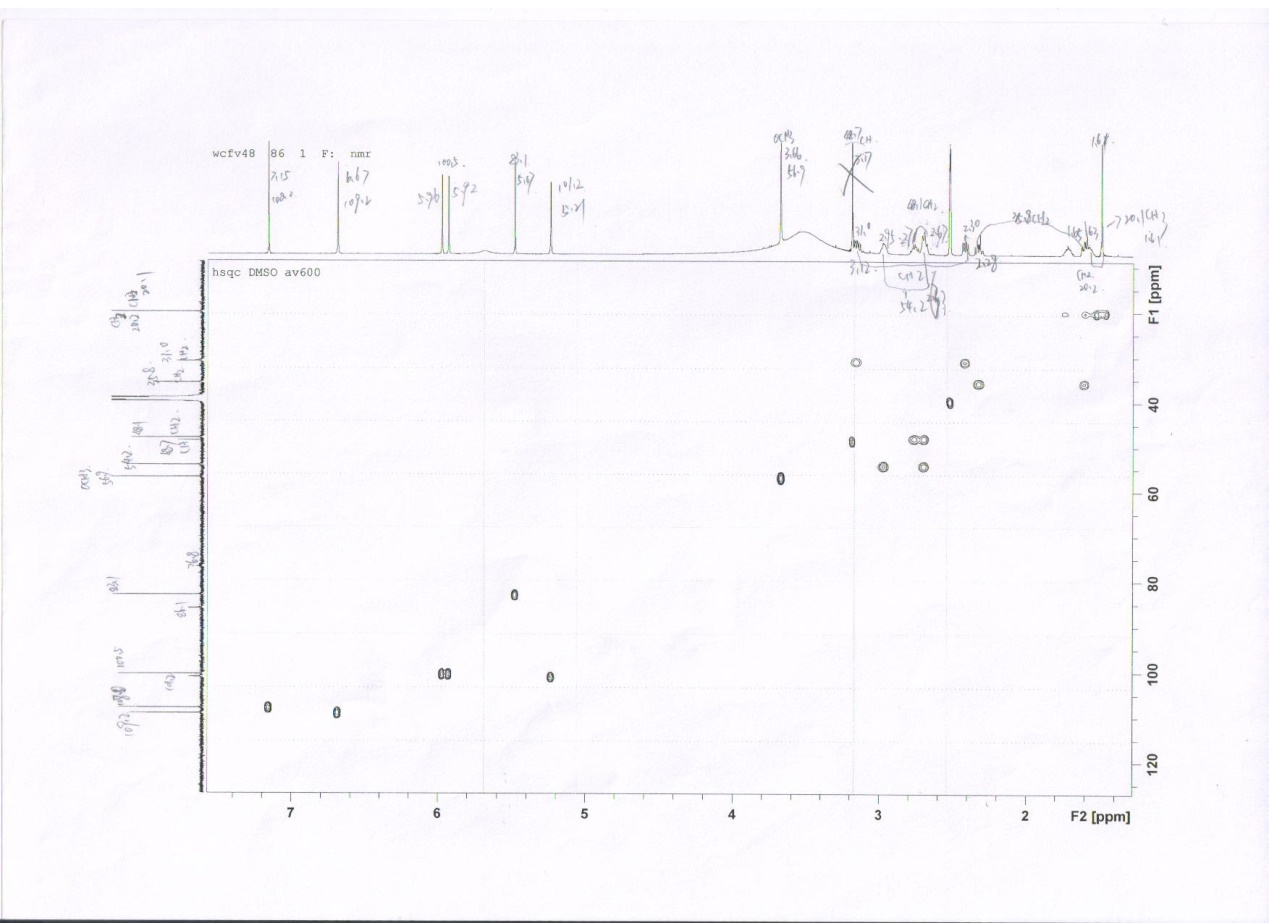


**Fig S20**. HSQC spectrum of **4** in acetone-*d*_6_.


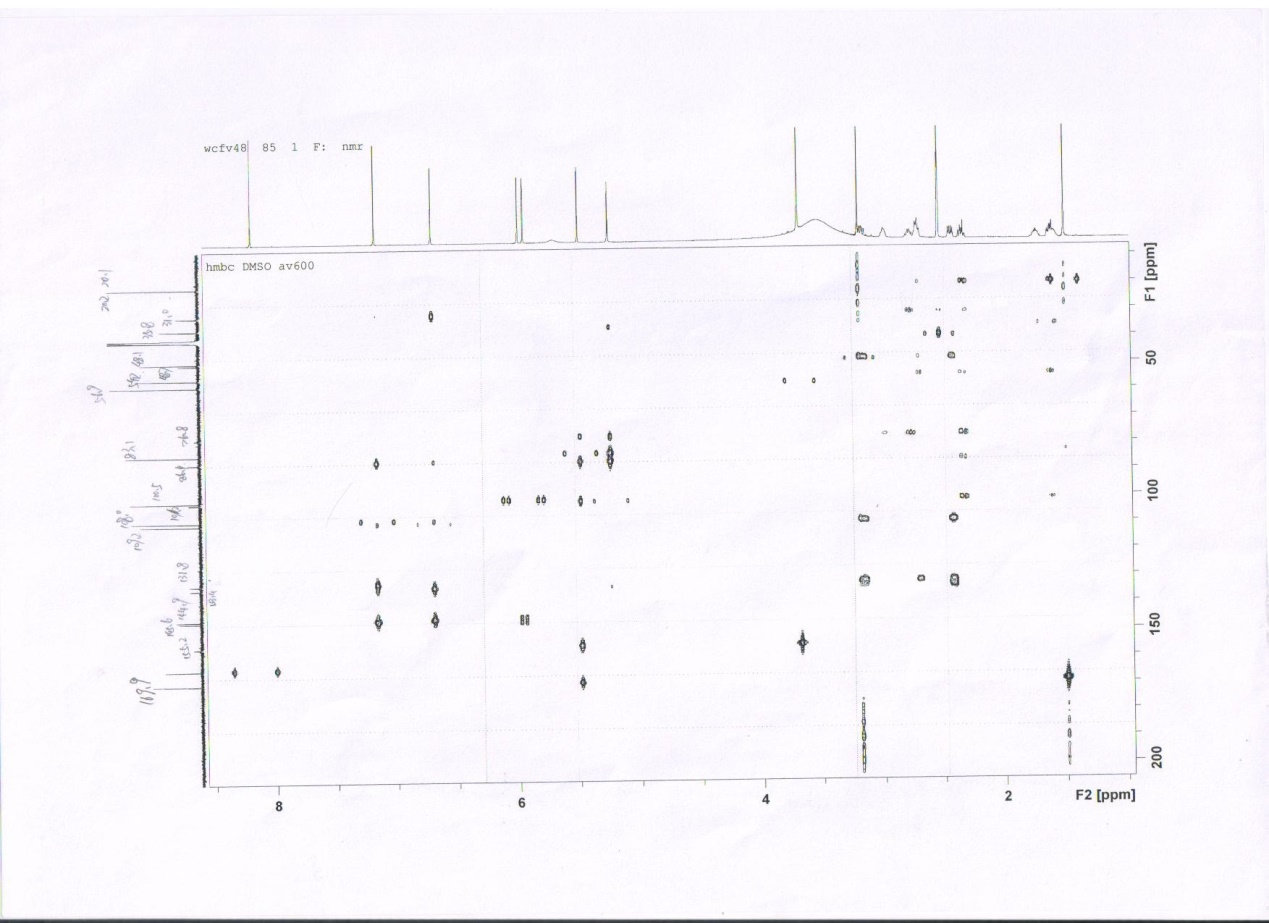


**Fig S21**. HMBC spectrum of **4** in acetone-*d*_6_.


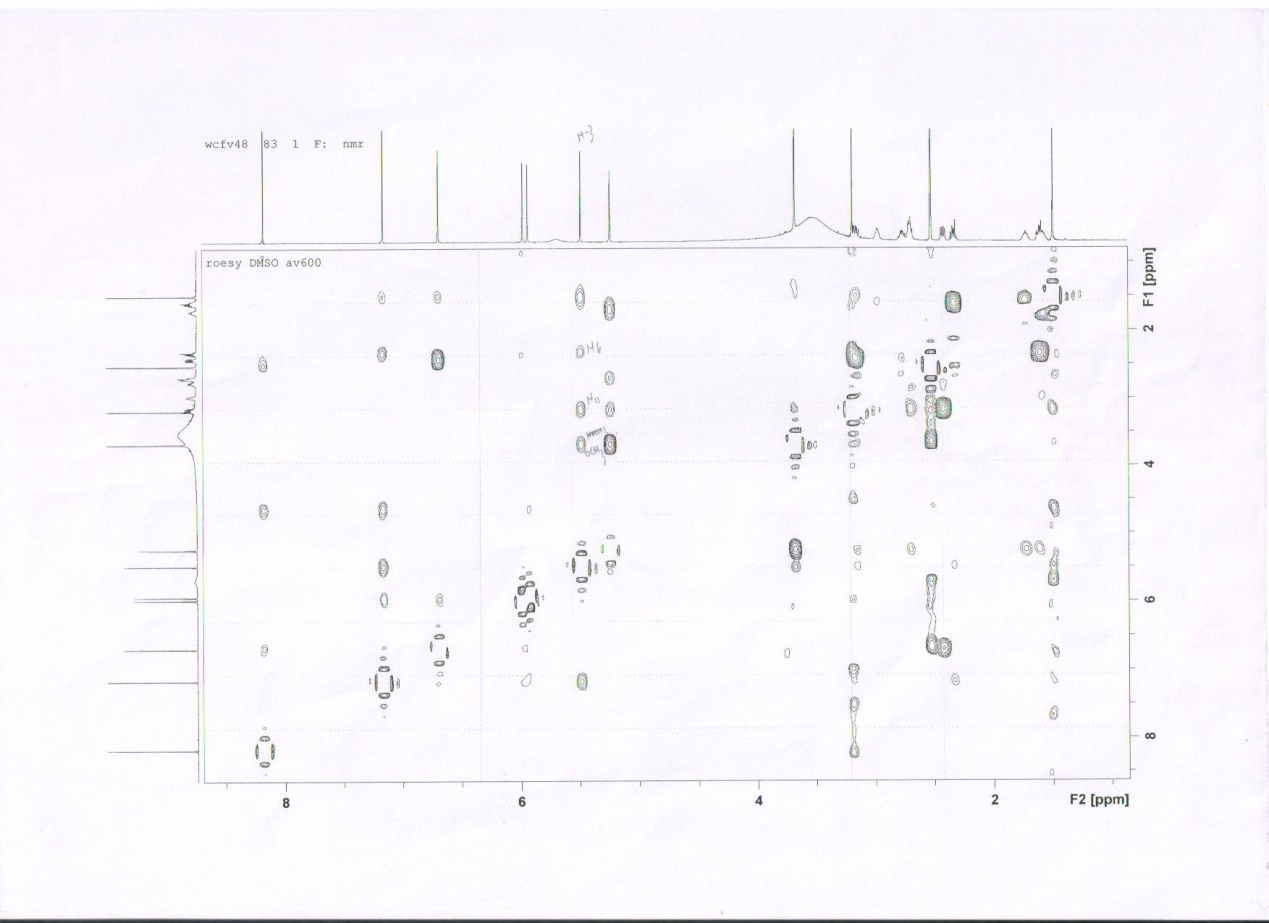


**Fig S22**. ROESY spectrum of **4** in acetone-*d*_6_.


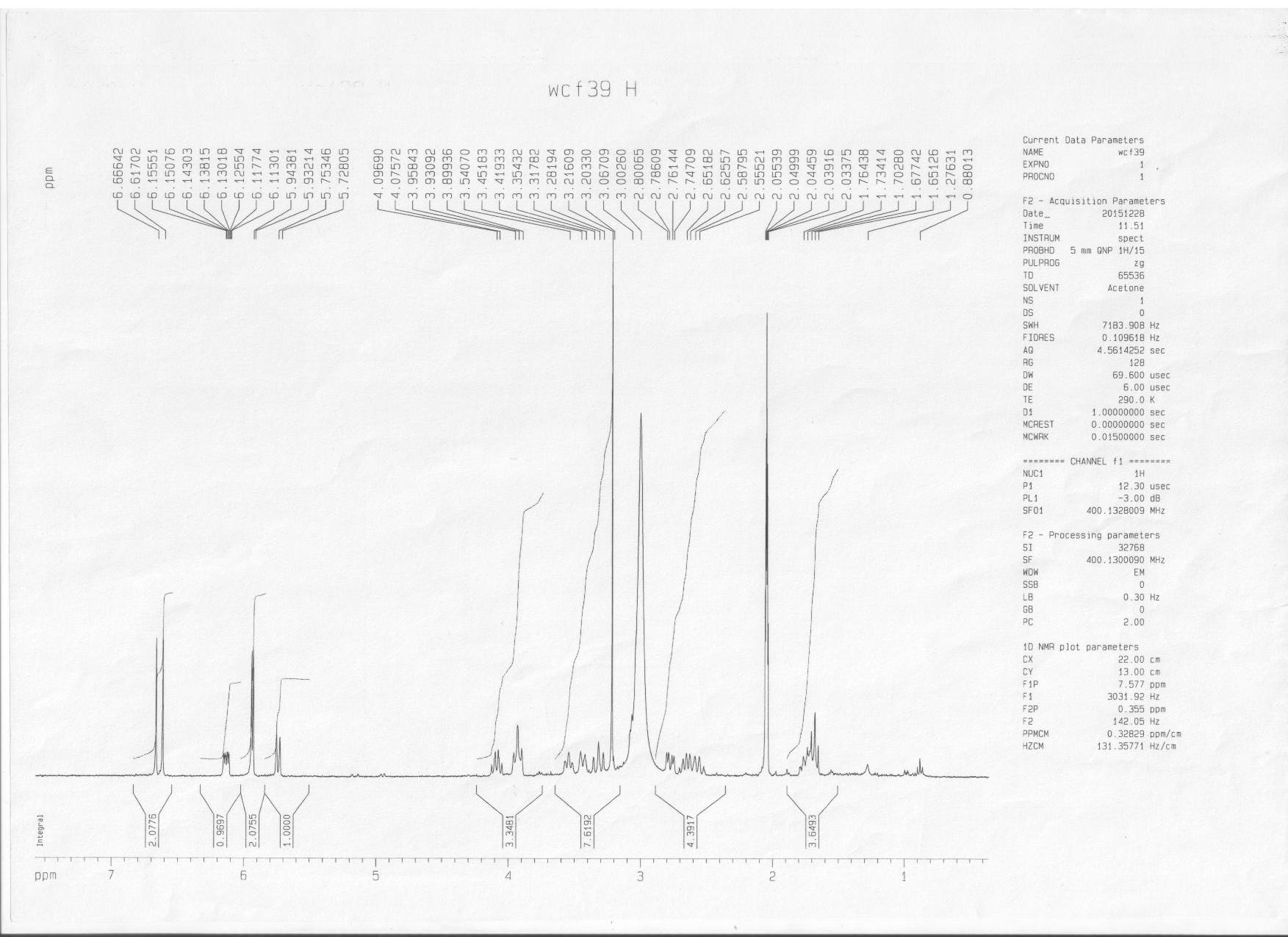


**Fig S23**. ^1^H NMR spectrum of **5** in acetone -*d_6_*.


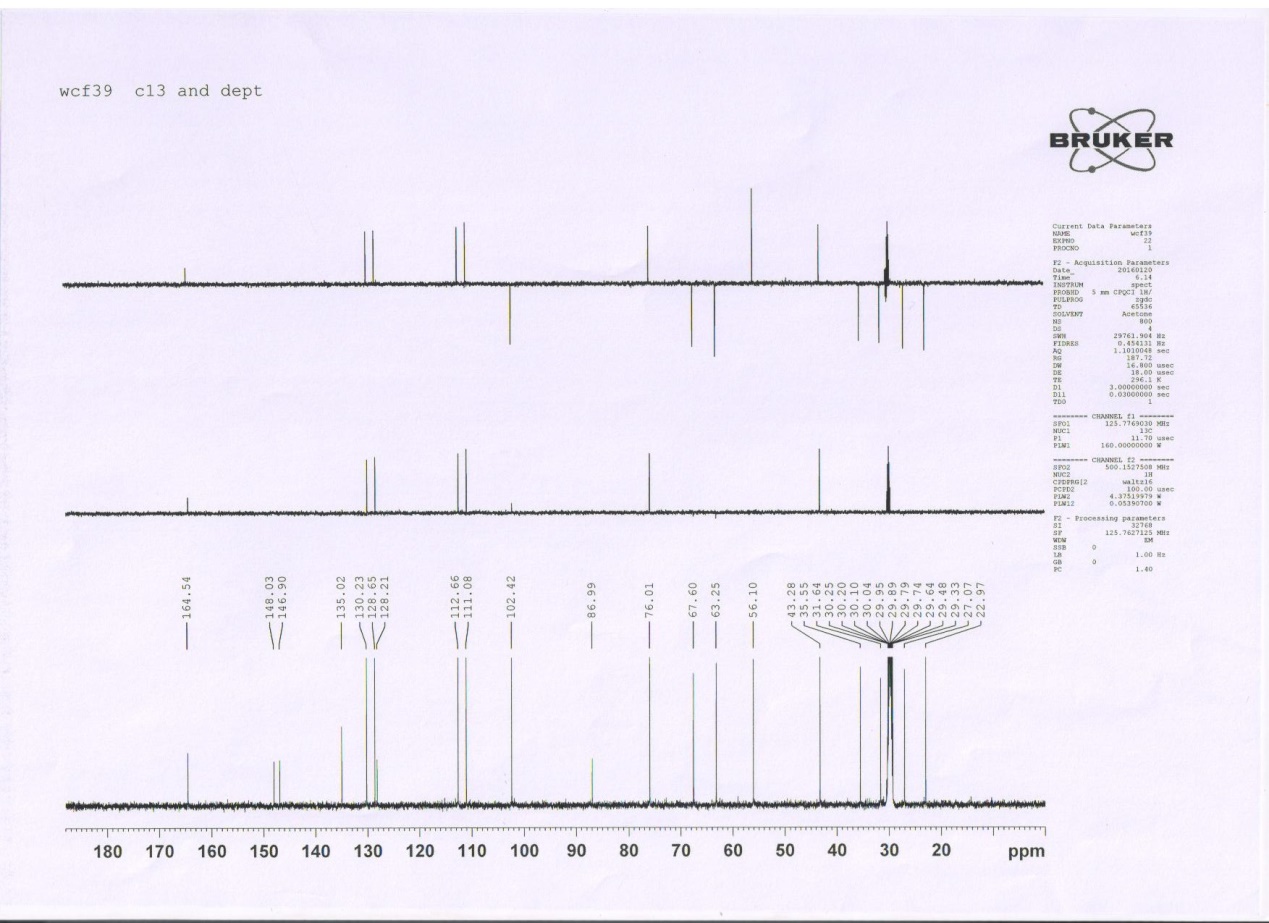


**Fig S24**. ^13^C and DEPT spectrum of **5** in acetone -*d_6_*.


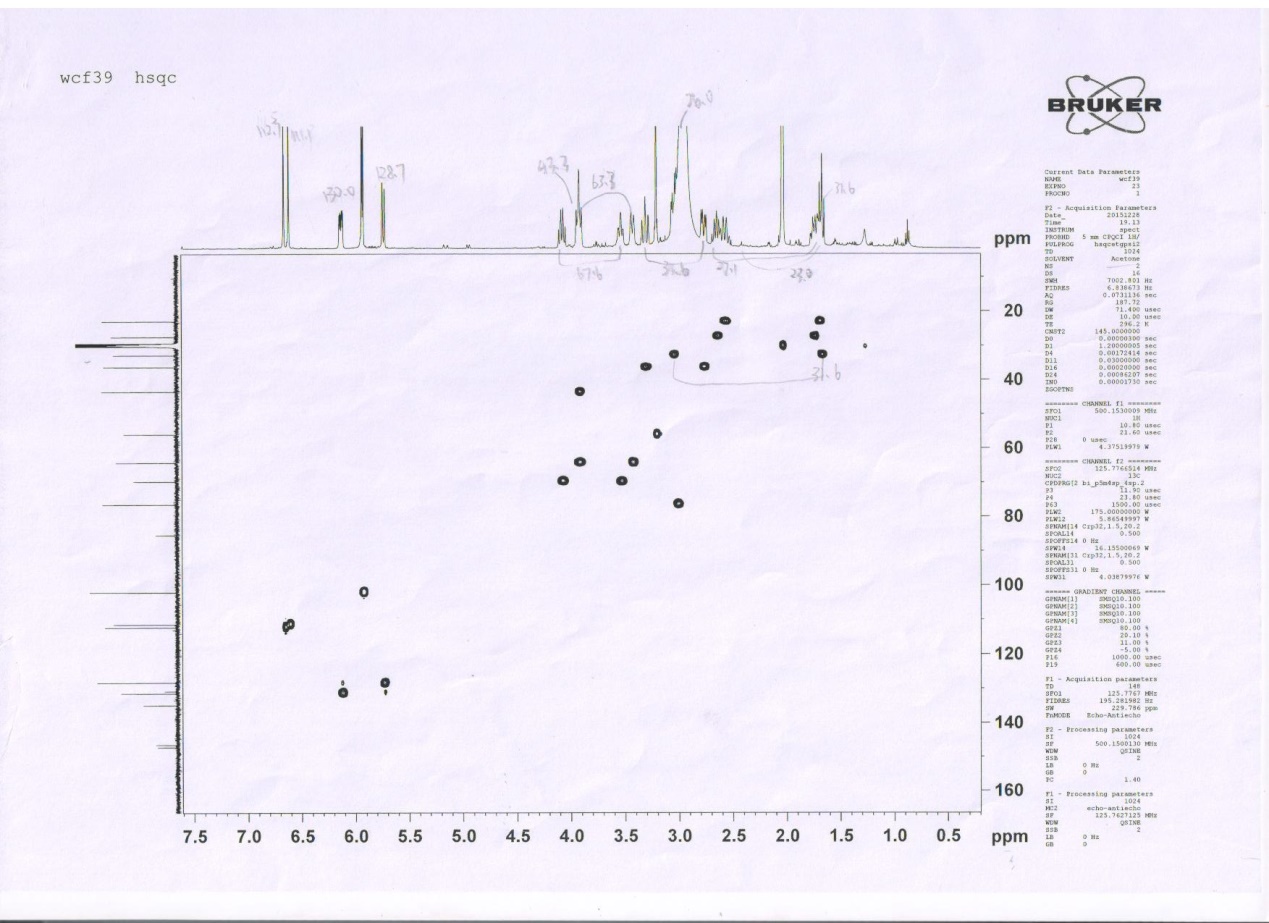


**Fig S25**. HSQC spectrum of **5** in acetone-*d*_6_.


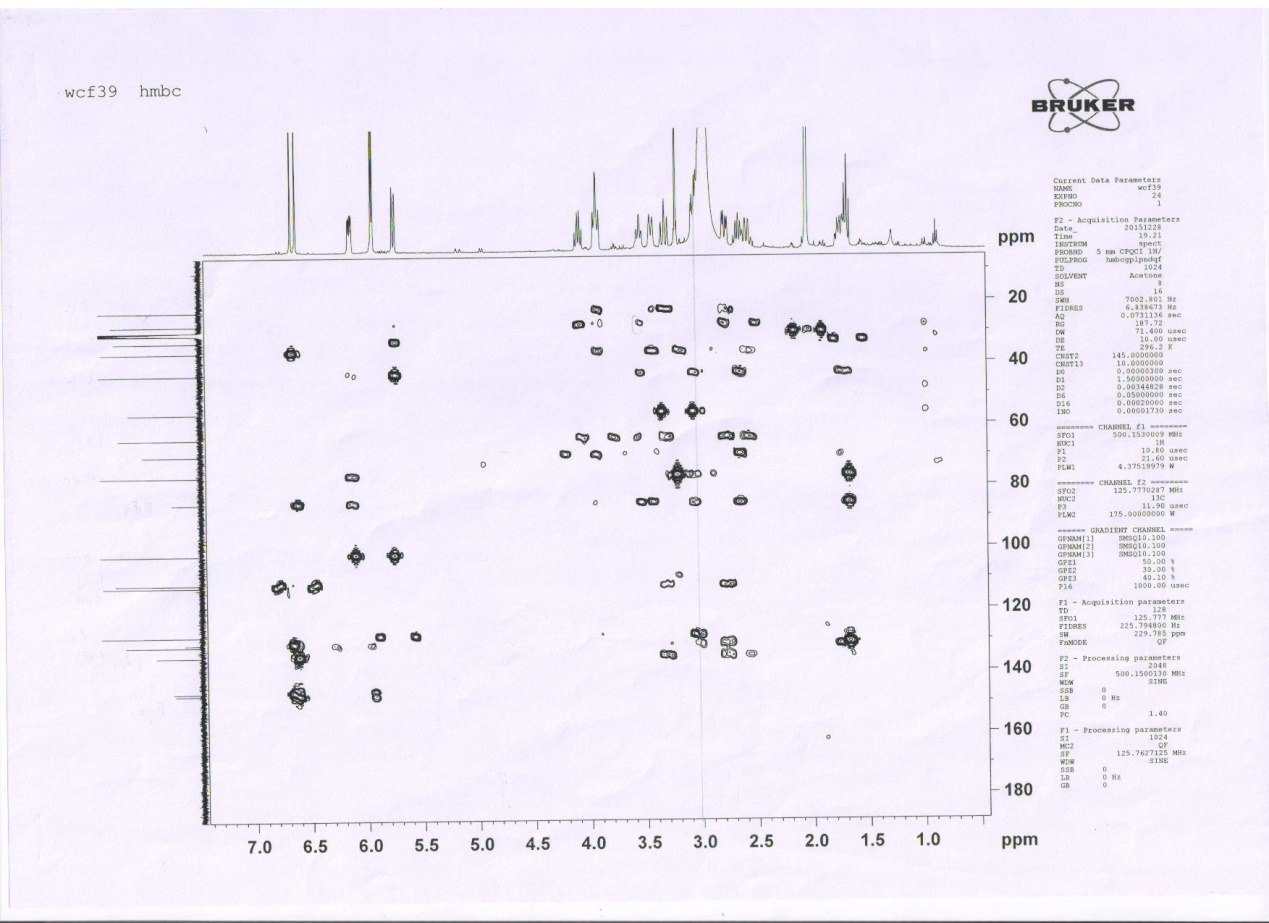


**Fig S26**. HMBC spectrum of **5** in acetone-*d*_6_.


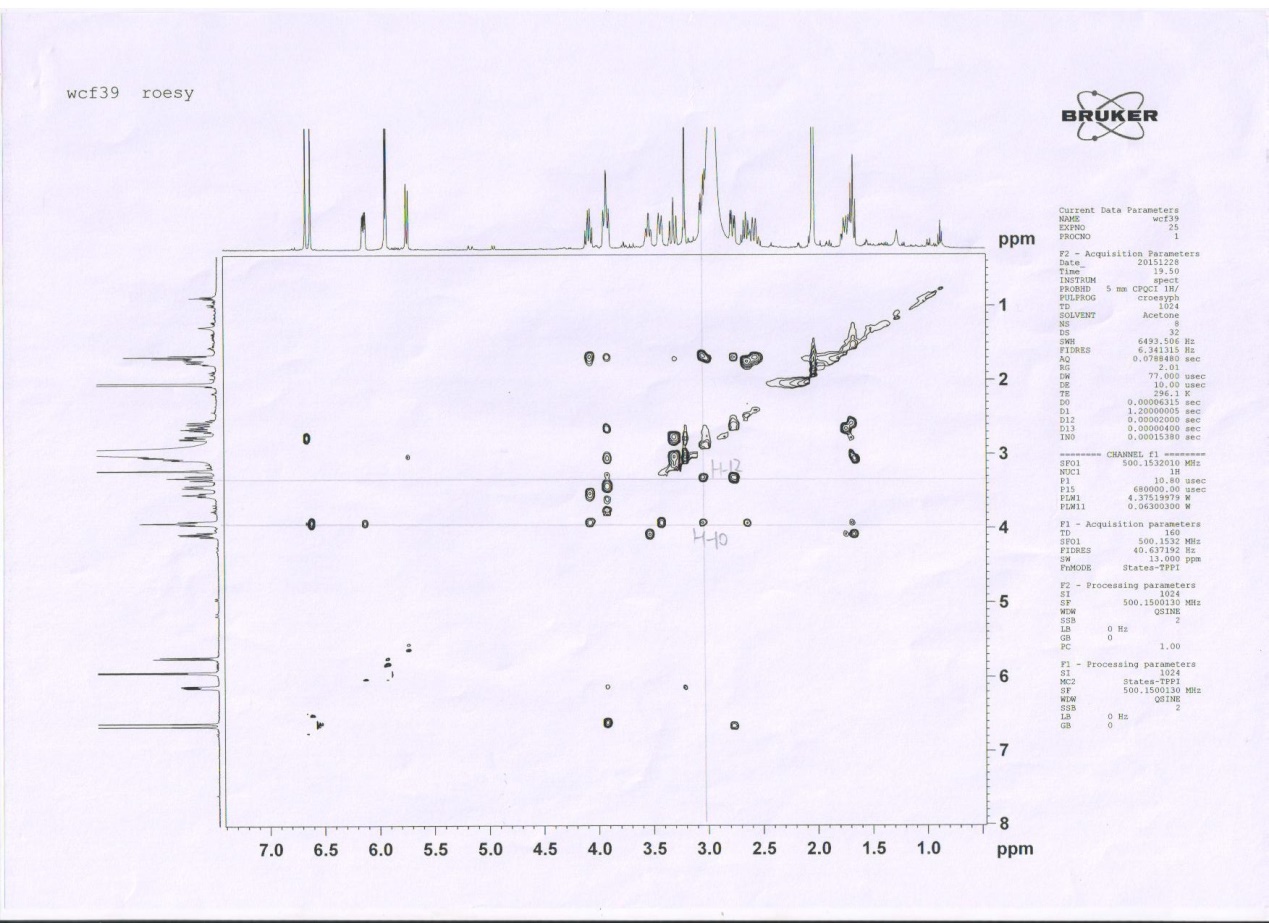


**Fig S27**. ROESY spectrum of **5** in acetone-*d*_6_.
